# Site-Selective Antibody Conjugation with Dibromopyrazines

**DOI:** 10.1021/acs.bioconjchem.4c00296

**Published:** 2024-08-16

**Authors:** Dénes Szepesi Kovács, Bettina Pásztor, Péter Ábrányi-Balogh, László Petri, Tímea Imre, József Simon, Enikő Tátrai, György Várady, József Tóvári, Peter A. Szijj, György M. Keserű

**Affiliations:** † Medicinal Chemistry Research Group, Research Centre for Natural Sciences, Magyar tudósok krt. 2, H-1117 Budapest, Hungary; ‡ Department of Organic Chemistry and Technology, Faculty of Chemical Technology and Biotechnology, Budapest University of Technology and Economics, Műegyetem rkp. 3, H-1111 Budapest, Hungary; § National Drug Research and Development Laboratory, Magyar tudósok krt. 2, H-1117 Budapest, Hungary; ∥ Institute of Chemistry, Faculty of Science, Eötvös Loránd University, Egyetem t. 1–3, H-1053 Budapest, Hungary; ⊥ MS Metabolomics Research Laboratory, Research Centre for Natural Sciences, Magyar tudósok krt. 2, H-1117 Budapest, Hungary; # Department of Experimental Pharmacology, National Institute of Oncology, Ráth György u. 7–9, H-1122 Budapest, Hungary; ∇ National Tumor Biology Laboratory, Ráth György u. 7–9, H-1122 Budapest, Hungary; ○ Molecular Cell Biology Research Group, Research Centre for Natural Sciences, H-1117 Budapest, Hungary; ◆ Department of Chemistry, University College London, WC1H 0AJ London, U.K.

## Abstract

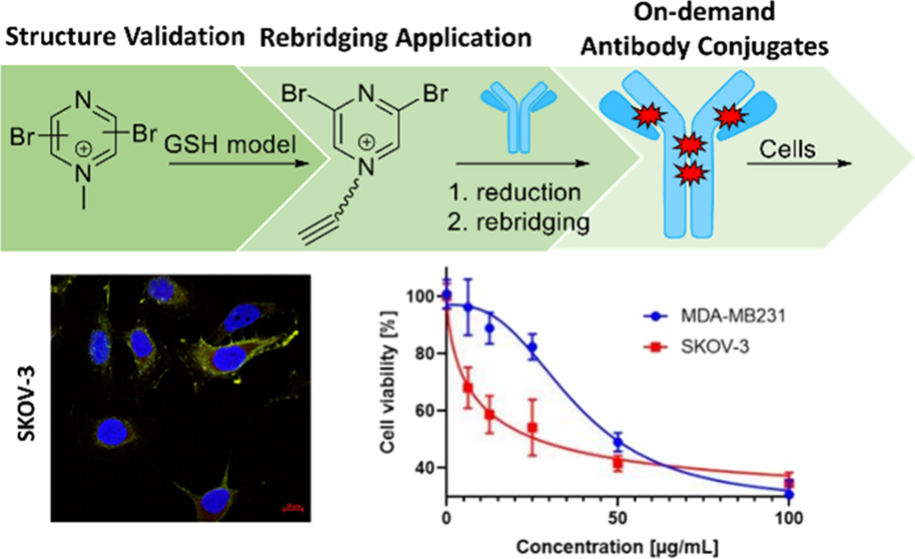

In recent years, antibody conjugates have evolved as state-of-the-art options for diagnostic and therapeutic applications. During site-selective antibody conjugation, incomplete rebridging of antibody chains limits the homogeneity of conjugates and calls for the development of new rebridging agents. Herein, we report a dibromopyrazine derivative optimized to reach highly homogeneous conjugates rapidly and with high conversion on rebridging of trastuzumab, even providing a feasible route for antibody modification in acidic conditions. Furthermore, coupling a fluorescent dye and a cytotoxic drug resulted in effective antibody conjugates with excellent serum stability and *in vitro* selectivity, demonstrating the utility of the dibromopyrazine rebridging agent to produce on-demand future antibody conjugates for diagnostic or therapeutic applications.

## Introduction

**Figure 1:**
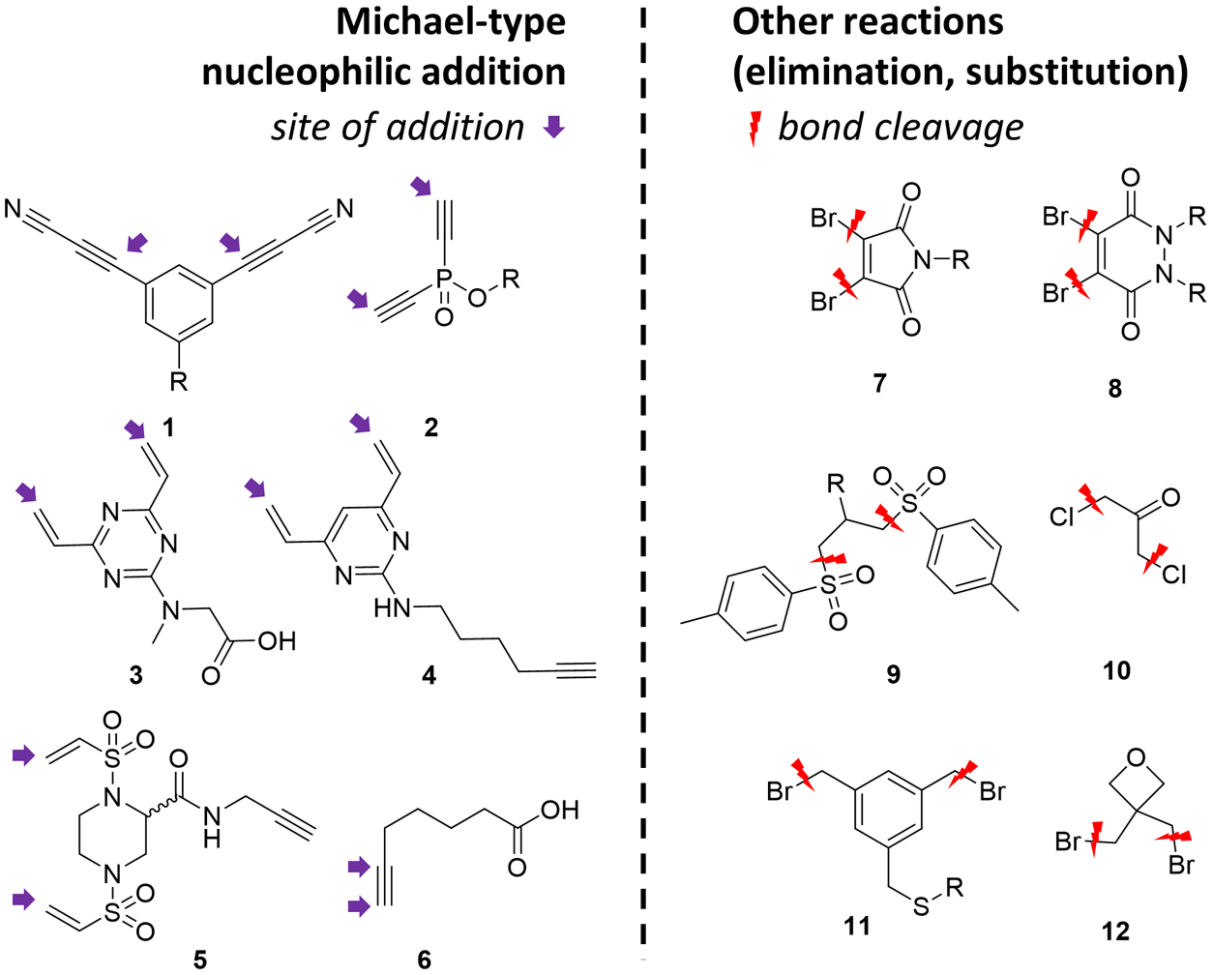
Rebridging agents with different mechanisms of action.

Target-specific antibodies are used extensively in cancer treatment and diagnosis to precisely deliver high-potency drugs, fluorescent dyes, or other markers to cancer cells.^1^ In fact, an increasing number of antibody–drug conjugates (ADCs) has been approved during the recent decade.^2^ However, many of these conjugates are produced by stochastic *N*-acylation techniques (such as Kadcyla and Besponsa) resulting in typically heterogeneous products that might be unfavorable from therapeutic and diagnostic standpoints.^3,4^ Therefore, several site-specific labeling methods emerged recently and the latest ADCs entering human trials were developed by these novel techniques.^5,6^ These strategies include the labeling of engineered cysteine residues^7^ or incorporating unnatural amino acids^8^ for biorthogonal reactions and reactive recognition tags^9^ that both require mutations of the native protein. The alternative enzymatic modification of glycans^10^ and amino acid side chains^11^ of the wild-type protein might lead to specificity problems. In contrast, the reduction of the solvent-accessible interchain disulfides followed by conjugating a bidentate reagent that can rebridge the chains provides a unique opportunity to label antibodies specifically while keeping their secondary and tertiary structures intact.^12^ These agents usually contain two thiol-reactive functional groups and a handle that can be equipped with a cytotoxic or fluorescent payload. This strategy effectively controls the number of conjugated small molecules due to structural restrictions, particularly, the four disulfide bridges enable 4 covalently attached small molecules resulting in the degree of labeling (DOL) being four in most cases. Recently, several rebridging agents were reported, differing in the mechanism of forming the sulfur–carbon bond (Figure 1). One subset of those acts in Michael-type addition (**1**–**6**),^13−18^ while other compounds are rebridging by different mechanisms including elimination (**7**–**9**)^12,19−22^ or nucleophilic substitution (**10**–**12**).^23−25^ The main disadvantage of the latter agents is the reversibility of the labeling reaction that might cause stability issues or require additional steps to avoid the unwanted nonspecific payload release. Both types of agents typically require complicated multistep syntheses and purification.

Considering that rebridging agents should react with thiols provided by the reduction of the interchain disulfide bonds, the ideal candidate should have (i) equal reactivity of the reacting bidentate electrophiles, (ii) small size to fit the interchain space, and (iii) a handle in an adequate position to functionalize the conjugate with diagnostic or therapeutic agents. To meet these requirements together with an easy and short synthesis from cheap reactants, we envisaged a *N*-quaternized heterocycle with two halogen warheads as a bidentate electrophile. The use of the S_N_Ar reaction would ensure irreversibility; the two halogens are small and symmetric and are expected to have similar reactivity. The single heterocyclic core might be small enough to keep the antibody fragments close, and the handle could be attached to the nitrogen atom in a simple reaction. Therefore, we first analyzed the l-glutathione (GSH) reactivity of our cysteine-selective heterocyclic fragments that suggested substituted pyridines and pyrazines as suitable starting points.^26−28^ Although some previous attempt was made for antibody modification with quaternized heterocyclic structures,^29^ no rebridging could have been achieved due to their single electrophile functions. 2,6-Dihalogenated pyridines seemed reasonably reactive in S_N_Ar reactions; however, installing the handle at position 1 between two neighboring halogens might have steric issues, while position 4 requires the formation of a new C–C bond. Instead, pyrazine quaternized at position 4 is a better option that would also ensure the appropriate reactivity toward cysteines.^26,30^ Therefore, the dibromopyrazine scaffold was a good choice, having two nitrogen atoms in the ring that activate the halogens positioned in the required symmetry and provide a straightforward option for quaternization.

In continuation of our interest in antibody modification,^31−34^ we aimed to develop a valuable, novel rebridging protocol. Thus, we have tested dibromopyrazinium derivatives and optimized the rebridging of trastuzumab, a clinically established antibody in the treatment of HER2-positive cancers.^35,36^ Next, using the optimized analogue, we evaluated possible off-target labeling reactions and confirmed the specificity of the generated antibody conjugate. Finally, we performed click reactions with a fluorescent dye and a cytotoxic drug and successfully tested the resulting conjugates *in vitro*. Our results suggest that dibromopyrazinium-based rebridging agents might be useful to generate on-demand ADCs or diagnostic tools in the future.

## Results and Discussion

### Synthesis and Characterization of Dibromopyrazines

**Scheme 1:**
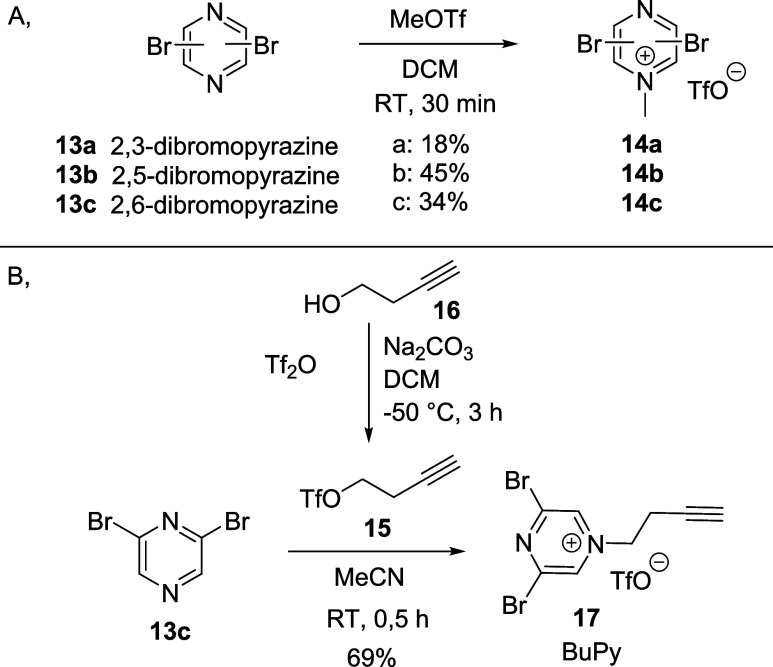
Synthesis of (A) *N*-methyl-dibromopyrazine Tool Compounds (**14a**–**c**) and (B) the New Rebridging Agent BUPY (**17**)

First, dibromopyrazines (**13a**–**c**) were quaternized at N-4 using methyl triflate (Scheme 1A) since alkylation by iodomethane was not successful. Next, we performed kinetic measurements by HPLC-MS to evaluate aqueous stability and reactivity toward GSH as a thiol surrogate (Table S2). 3,5-Dibromopirazinium **14c** reacted with GSH immediately and formed the corresponding pyrazinium-bridged dimer of GSH. We have also investigated 2,3- (**14a**) and 2,5-dibromopyraziniums (**14b**); however, these derivatives reacted with water immediately instead of GSH. Therefore, we concluded that **14c** would be the ideal core of the novel rebridging agent. This core was equipped with a clickable handle by incorporating an acetylene group using triflate **15** formed from but-3-yne-4-ol (**16**). The reaction went smoothly in 30 min at room temperature providing 3,5-dibromo-1-(but-3-ynyl)pyrazin-1-ium triflate (**17**, BUPY) in a good yield (69%, Scheme 1B). Next, we evaluated the aqueous stability of BUPY (**17**) in borate-buffered saline (BBS) buffer (pH = 8) and its reactivity against free thiols using GSH similar to its predecessor **14a**.^37^ The reaction with GSH showed full conversion in less than 5 min, and the half-life in the buffer was more than 5 h (Table S2).

### Development of the Antibody Rebridging Agent (BUPY)

**Figure 2:**
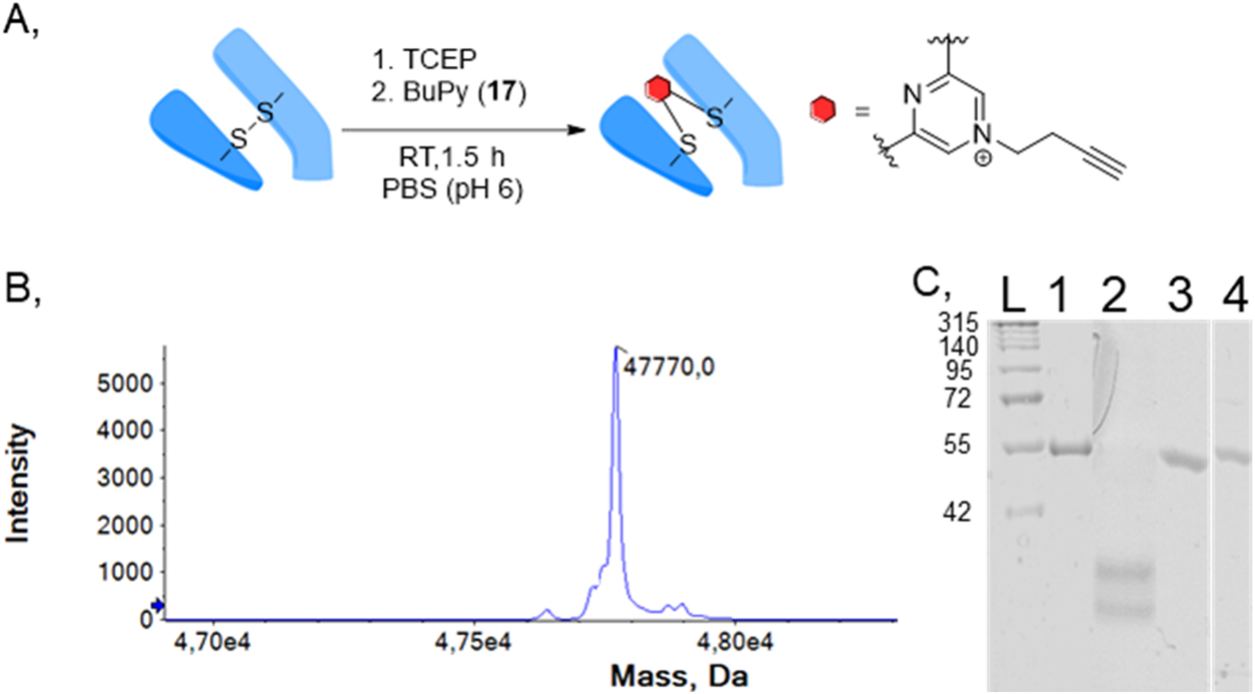
Rebridging of reduced Fab_HER2_ with BUPY (**17**). (A) Rebridging scheme. (B) Deconvoluted MS spectrum of Fab_HER2_-BUPY (expected: 47 771 Da, observed: 47 770 Da). (C) SDS-PAGE of Fab_HER2_-BUPY: L: protein ladder; 1: native Fab_HER2_; 2: reduced Fab_HER2_; 3: rebridged Fab_HER2_-BUPY; 4: rebridged Fab_HER2_-BUPY by reducing conditions.

**Figure 3:**
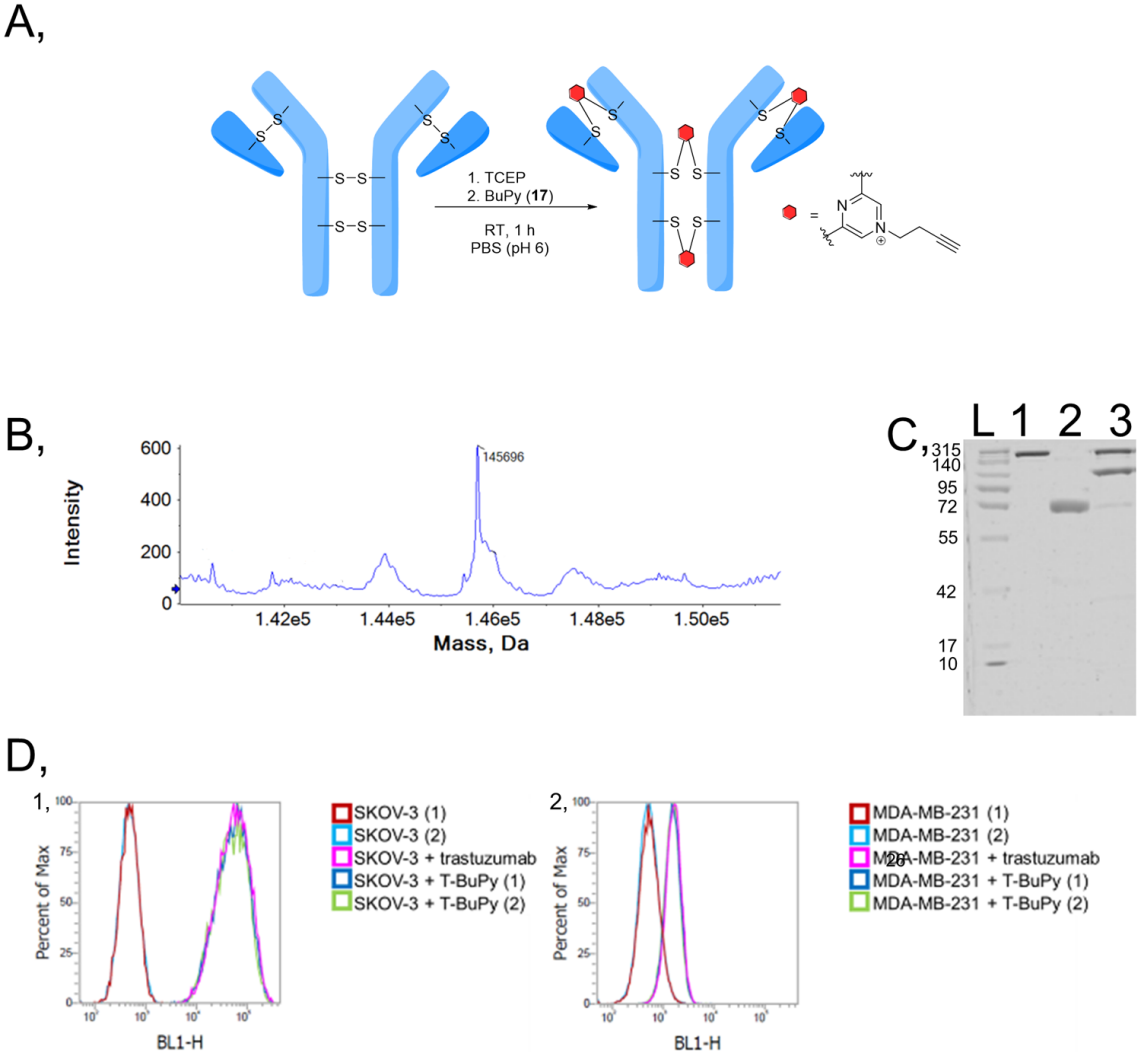
(A) Synthesis of trastuzumab-BUPY antibody conjugate; (B) MS spectrum of trastuzumab-BUPY antibody conjugate (expected: 145 694 Da, observed: 145 697 Da); (C) SDS-PAGE of trastuzumab-BUPY antibody conjugate (L: protein ladder; 1: native trastuzumab; 2: reduced trastuzumab; 3: trastuzumab-BUPY). (D) Flow cytometry analysis of trastuzumab-BUPY: 1. SKOV-3 (HER2^hi^) cell line treated with native and BUPY-modified trastuzumab; 2. MDA-MB-231 (HER2^lo^) cell line treated with native and BUPY-modified trastuzumab.

To develop a readily available rebridging method by applying dibromopyrazine **17**, we aimed to optimize the rebridging reaction. First, reduced Fab_HER2_ was treated with BUPY (**17**) following a stepwise method^38^ (Figure 2A) at different pH levels (Figure S1). After 90 min, the conjugates were examined by SDS-PAGE (Figure S1A) and UHPLC-MS. We obtained the optimal DOL = 1 by mass spectrometry (Figures 2B and S1) and SDS-PAGE (Figure 2C) and showed full conversion of the rebridging at pH = 6. Next, we examined the cysteine selectivity with native Fab_HER2_. BUPY (**17**) was added to Fab_HER2_ without TCEP. The protein thus contained no free thiols, and the reactivity of BUPY (**17**) with other nucleophilic residues (lysine, serine, etc.) could be examined. The mixture was incubated at 37 °C with constant agitation for 90 min, and no modified Fab_HER2_ was detected (Figure S2). Next, we performed a follow-up click reaction to demonstrate the readily available functionalization of rebridged Fab_HER2_-BUPY with azido-SMCC-DM1 (**18**). According to UV spectroscopy and MS (Figure S3) analysis, the click reaction went pleasingly and resulted in the loading of 1 cytotoxic drug. We aimed to use the same stepwise strategy for the rebridging of full trastuzumab (Figure 3A) and to optimize the reaction parameters including the excess of BUPY, antibody concentration, and incubation time. To produce antibody conjugates with a high conversion rate (>95%), the optimal ratio was found to be 10:1 BUPY (**17**) to trastuzumab. Next, we further optimized the protocol by studying the antibody concentration and reaction time. We found DOL = 4 conjugate was formed even at low concentrations (5 μM) with minimal changes on the SDS gel (Figure S4). The reaction reached full conversion in 1 h at all antibody concentrations (5, 10, and 20 μM). These results suggest that optimal conditions of rebridging are 10 equiv BUPY (**17**), PBS (phosphate-buffered saline) pH 6.0, room temperature, and 1 h incubation. As known rebridging agents work mostly in the basic pH range,^15,16,39^ BUPY could provide a valuable alternative to those, enabling antibody rebridging efficiently even at slightly acidic conditions, which is advantageous, because antibody conjugates are generally more stable around pH = 6, therefore the storage buffers are often also acidic.^40,41^ In conclusion, we confirmed the utility of *N*-quaternized dibromopyrazine scaffold as a novel antibody rebridging agent, resulting in DOL = 4 antibody conjugate with high rebridging (97 ± 2%) conversion (Figure 3B,C).

**Figure 4:**
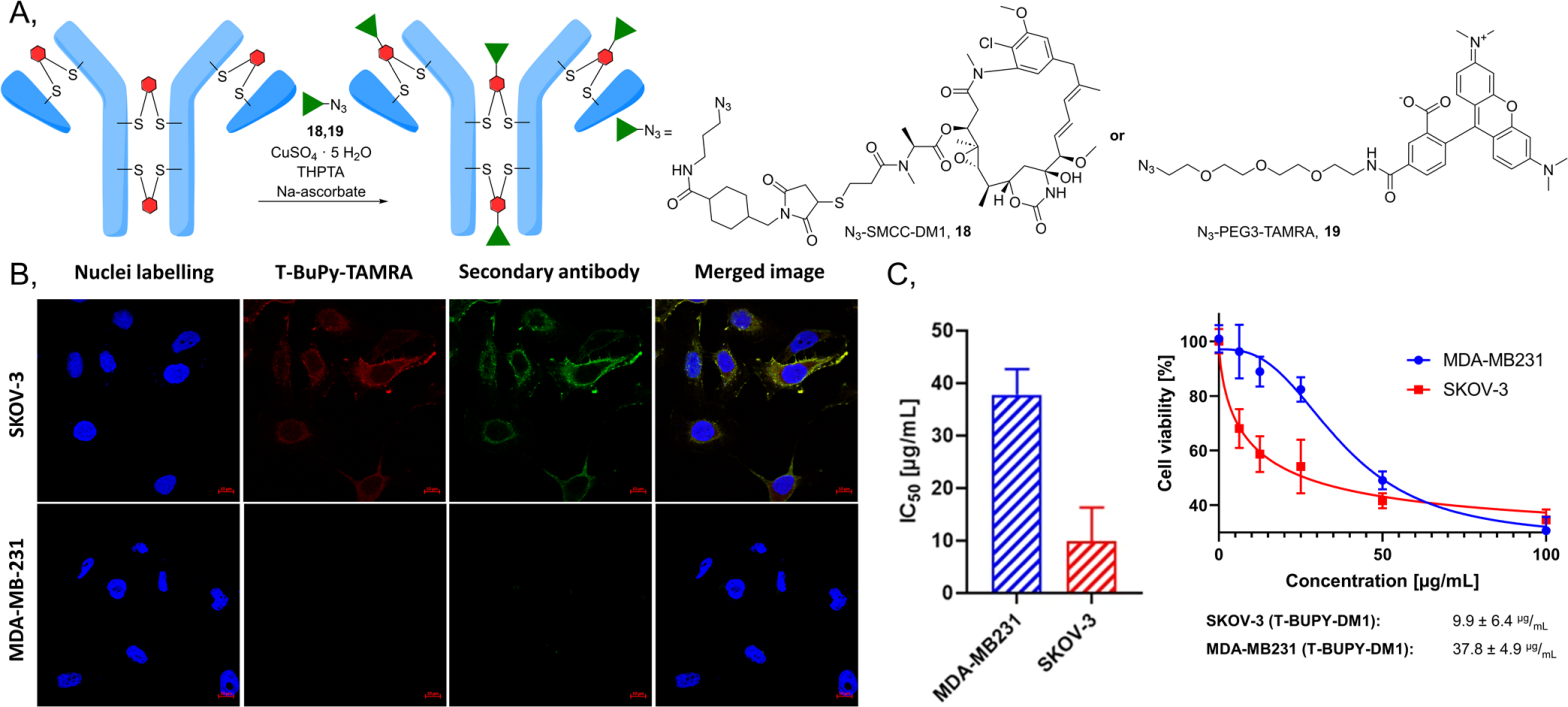
Biological evaluation of BUPY antibody conjugates. (A) Click reaction of trastuzumab-BUPY with the cytotoxic drug (**18**) and fluorescent dye (**19**) azides. (B) Trastuzumab-BUPY-TAMRA immunostaining on SKOV-3 HER2^hi^ and MDA-MB-231 HER2^lo^ cell lines. The nuclei of the cells are in blue, and trastuzumab-BUPY-TAMRA staining is shown in red. The FITC-conjugated goat antihuman IgG secondary antibody is visible in green. Based on the images, the staining is selective for HER2^hi^ cells and located on the cell surfaces in the case of SKOV-3 cells as expected. Scale: 10 μm. (C) *In vitro* cell viability of SKOV-3 and MDA-MB-231 cells incubated with trastuzumab-BUPY-DM1 cytotoxic ADC.

Finally, we challenged the retained biological activity of the modified antibody and successfully proved the selective recognition of relevant cancer cells using flow cytometry differentiating between SKOV-3 (HER2^hi^) and MDA-MB-231 (HER2^lo^) cell lines (Figure 3D). This encouraged us to use BUPY (**17**) as a rebridging agent for functionalized ADCs. We performed click reactions with the fully rebridged antibody (Figure 4A), applying the same protocol as that for the successful Fab_HER2_-BUPY click reaction. We used azido-SMCC-DM1 (**18**, Scheme S1) to produce a therapeutic conjugate and azido-PEG3-TAMRA (**19**) as a diagnostic conjugate. The conjugates were examined by UV spectroscopy (Figures S6 and S7), giving a loading of 4 on average for both, and moreover, a fluorescent signal was also detected on SDS-PAGE in the case of trastuzumab-BUPY-TAMRA (Figure S8). After the successful conjugation of therapeutic agent DM1 and fluorescent dye TAMRA, we initiated *in vitro* biology experiments and investigated the stability of the conjugate. To visualize the selectivity of the dye-conjugated antibody, we treated SKOV-3 HER2 overexpressing cells and MDA-MB-231 as control cell lines with trastuzumab-BUPY-TAMRA. Confocal microscopy showed significant membrane labeling for SKOV-3 cells (red contour, Figure 4B), while no membrane labeling was observed for HER2^lo^ cells (MDA-MB-231, Figure 4B). Also, these results were further confirmed by FITC-labeled secondary antibody labeling (green contour, Figure 4B). Based on the localization of the signal and the significantly lower immunostaining in the control cell line, we concluded that the conjugate was selective and sufficiently sensitive toward HER2^hi^ cells. After these results, we examined the stability of the BUPY-rebridged antibody conjugate. Trastuzumab-BUPY-TAMRA was incubated in bovine serum with added GSH (total concentration of 1 μM) at 37 °C for 7 days to mimic *in vivo* conditions.^24^ The Coomassie staining and in-gel fluorescence showed that trastuzumab-BUPY-TAMRA remained intact. In particular, the fluorescent dye was not transferred onto serum proteins, and individual heavy or light chains were not released from the rebridged antibody (Figure S9). Finally, the cytotoxic conjugate was submitted to an *in vitro* cell viability assay on SKOV-3 (HER2^hi^) and MDA-MB-231 (HER2^lo^) cells. The results revealed that the cytotoxic effect was significantly increased on the HER2^hi^ cell line compared with the HER2^lo^ control (9.9 ± 6.4 and 37.8 ± 4.9 μg/mL, respectively; Figure 4C).

## Conclusions

We developed BUPY, a novel disulfide rebridging agent produced in a simple and fast reaction using safe and inexpensive reagents. Our results confirmed its excellent aqueous stability, appropriate cysteine reactivity, and high target selectivity. We successfully performed a Fab rebridging and click reaction with the cytotoxic payload (DM1) of a commercialized ADC (Kadcyla). Monoclonal antibody rebridging with BUPY resulted in DOL = 4 conjugates. BUPY-based modification of the antibody did not influence the selectivity of trastuzumab as the rebridged antibody bound to HER2^hi^ cells selectively according to flow cytometry measurements. Furthermore, the effective syntheses of diagnostic and therapeutic conjugates suggest the on-demand applicability of BUPY for ADCs. With *in vitro* experiments, we proved the stability, selectivity, and efficacy of the antibody conjugates. Thus, BUPY presented herein can be an easily accessible and highly effective rebridging agent for the development of future ADCs.

## Supporting Information Available

Experimental procedures and additional figures, SDS-PAGE gel images, photophysical measurements, HPLC-MS kinetics measurements, LC-MS measurements of antibody conjugates, biological measurements (including cell line and culture conditions, flow cytometry, immunocytochemistry, and *in vitro* cell viability MTT assay), synthetic procedures, and ^1^H and ^13^C NMR spectra of novel compounds (PDF)
