# Supplementary material for: Site-Selective Antibody Conjugation with Dibromopyrazines
Source: Bioconjug Chem. 2024 Aug 16;35(9):1373–9. doi: 10.1021/acs.bioconjchem.4c00296 (PMC11417993; doi:10.1021/acs.bioconjchem.4c00296)
Supplement: Supplementary file 1 — bc4c00296_si_001.pdf [file bc4c00296_si_001.pdf]

Supporting Information

Site-selective antibody conjugation with dibromopyrazines

Dénes Szepesi Kovács,[a,b,c] Bettina Pásztor,[a,b,c] Péter Ábrányi-Balogh,[a,b,c] László Petri,[a,c,d] Tímea Imre,[a,e] József Simon,[a,e] Enikő Tátrai,[f,g] György Várady,[h] József Tóvári,[f,g] Peter A. Szijj,[i] György M. Keserű*[a,b,c]

a. Medicinal Chemistry Research Group, Research Centre for Natural Sciences, Magyar tudósok krt. 2., H-1117 Budapest, Hungary
b. Department of Organic Chemistry and Technology, Faculty of Chemical Technology and Biotechnology, Budapest University of Technology and Economics, Műegyetem rkp. 3., H-1111 Budapest, Hungary
c. National Drug Research and Development Laboratory, Magyar tudósok krt. 2., H-1117 Budapest, Hungary
d. Institute of Chemistry, Faculty of Science, Eötvös Loránd University, Egyetem t. 1-3., H1053 Budapest, Hungary
e. MS Metabolomics Research Laboratory, Research Centre for Natural Sciences, Magyar tudósok krt. 2., H-1117 Budapest, Hungary
f. Department of Experimental Pharmacology, National Institute of Oncology, Ráth György u. 7-9., H-1122 Budapest, Hungary
g. National Tumor Biology Laboratory, Ráth György u. 7-9., H-1122 Budapest, Hungary
h. Molecular Cell Biology Research Group, Research Centre for Natural Sciences H-1117 Budapest, Hungary
i. Department of Chemistry, University College London, WC1H 0AJ London, UK

Table of contents

Experimental 2
General 2
SDS-PAGE and densitometry 2
Photophysical measurements 3
HPLC-MS kinetics measurements 3
LC-MS measurements of antibody conjugates 4
Biological measurements 5
Cell lines and culture conditions 5
Flow cytometry 5
Immunocytochemistry 6
MTT assay 7
Synthetic procedures 7
Results of HPLC-MS kinetic measurements 10
Trastuzumab Fab and mAb conjugation methods 10
Trastuzumab Fab generation 10
Fab_{HER2} rebridging with BUPY 11
Examining the selectivity of BUPY 11
Trastuzumab rebridging with BUPY 13
Click reaction of 5-TAMRA and T-BUPY conjugate 15
NMR spectra 18
Supplementary references 23

Experimental

General

Chemical reagents were purchased from Sigma Aldrich, Thermo Fisher and VWR International. Trastuzumab antibody was from Genentech Inc. The reactions were followed by analytical thin layer chromatography on silica gel 60 F254 and HPLC–MS chromatography with a Shimadzu LCMS-2020 device using a Reprospher 100 C18 (5 µm; 100 × 3 mm) column and positive-negative double ion source (DUIS) with a quadrupole MS analyzer in a range of 50–1000 m/z. High resolution mass spectra were recorded on a Waters Q-TOF Premier mass spectrometer in positive ESI ionization mode. ^{1}H and ^{13}C NMR spectra were recorded in $CDCl_3$ or DMSO-d_6 solution at room temperature, on a Varian Unity Inova 500 spectrometer (500 and 125 MHz for ^{1}H and ^{13}C/APT NMR spectra, respectively), and on a Varian Unity Inova 300 spectrometer (300, 75 MHz and 282 MHz for ^{1}H, APT NMR spectra, respectively), with the deuterium signal of the solvent as the lock and TMS as the internal standard. Chemical shifts (δ) and coupling constants (J) are given in ppm and Hz, respectively. All melting points were determined on a Jasco SRS OptiMelt apparatus (Stanford, CA, USA) and are uncorrected.
The antibody solutions were centrifuged with Eppendorf Centrifuge 5810 R.

SDS-PAGE and densitometry

Non-reducing glycine-SDS-PAGE at 10% acrylamide running were performed following standard lab procedures. A 4% stacking gel was used and a broad-range MW marker (4.6–300 kDa, ProSieve QuadColor Protein Marker, Lonza) was co-run to estimate protein weights. Samples (10 µL at 5 µM) were mixed with loading buffer (3 µL, composition for 6×SDS: 1 g SDS, 3 mL glycerol, 6 mL 0.5 M Tris buffer pH = 6.8, 2 mg Coomassie-blue R250 in 10 mL), heated at 65 °C for 5 minutes. Samples were subsequently loaded into the wells in a volume of 13 µL. All gels were run at constant 200 mA for 45 minutes. Gels were stained using a Coomassie stain (0,12 g Coomassie-blue G-250, 0,10 g Coomassie-blue R-250, 500 mL MeOH, 400 mL distilled water, 100 mL acetic acid), after washing it was rested at room temperature for 16 h in water-ethanol mixture. Then the gels were imaged using HP Laserjet 1132 MFP scanner at 600 dpi. Images were saved under default brightness, contrast, and gamma settings. Densitometry was performed using ImageJ. Background subtraction was achieved using the built-in plugin with a rolling ball radius of 30, sliding paraboloid, and smoothing. Brightness and contrast settings were auto adjusted within the software.

Photophysical measurements

Absorbance measurements were carried out on a Jasco V-700 spectrophotometer (standard cell quartz cuvette with 1 cm light path length, 1 nm bandwidth, 400 nm/min recording speed) operating at 21 °C. We measured the absorbance spectra from 220 nm to 750 nm. The UV/Vis absorbance measurements of trastuzumab conjugate were carried out on Thermo Scientific NanoDrop ™ 1000 Spectrophotometer and SpectraMax iD5 Multi-Mode Microplate Reader (Molecular Devices; San Jose, CA). Before the measurements buffer exchange was performed six times with Sartorius Vivaspin 500 10000 MWCO at 15000 g for 10 minutes each time. Sample buffer was used as blank for baseline correction with extinction coefficients; ε_{252}= 81924 $M^{-1}cm^{-1}$ and ε_{330}= 0 $M^{-1}cm^{-1}$ for trastuzumab (in the equation T); ε_{252}= 1420 $M^{-1}cm^{-1}$for ε_{330}= 3828 $M^{-1}cm^{-1}$ for 3,5-dibromo-1-*N*-but-3-yn-1-yl-pyrazin-1-ium (in the equation BUPY) scaffold measured in BBS. DOL values were calculated from Lambert-Beer equation for absorbance at 252 and 330 nm.

$$A_{252\,nm} = \varepsilon_{BuPy,252\,nm} \cdot l \cdot c_{BuPy} + \varepsilon_{T,252\,nm} \cdot l \cdot c_T$$

$$A_{330\,nm} = \varepsilon_{BuPy,330\,nm} \cdot l \cdot c_{BuPy} + \varepsilon_{T,330\,nm} \cdot l \cdot c_T$$

From the concentration values the average DOL value was determined (c_{BUPY}/c_T) by CF=0.37.

$$DAR = \frac{\frac{A_{330\,nm}}{\varepsilon_{BuPy,330\,nm}}}{\frac{A_{252\,nm} - CF \cdot A_{330\,nm}}{\varepsilon_{trastuzumab,252\,nm}}}$$

HPLC-MS kinetics measurements

For GSH assay 500 μM solution of the probe (BBS buffer pH 8.0, 10 % acetonitrile, 250 μL) with 200 μM solution of indoprofen as internal standard was added to 10 mM glutathione solution (dissolved in BBS buffer, 250 μL) in 1:1 ratio. The final concentration was 250 μM **14** pyrazin-1-ium, 100 μM indoprofen, 5 mM glutathione and 5% acetonitrile (500 μL). The final mixture was analyzed by HPLC-MS after 0, 1, 2, 4, 8, 12, 24 h time intervals. Degradation kinetics were also investigated respectively using the previously described method, applying pure BBS buffer instead of the GSH-solution. In this experiment, the final concentration of the mixture was 250 μM fragment, 100 μM indoprofen and 5% acetonitrile. The AUC (area under the curve) values were determined *via* integration of HPLC spectra then corrected with internal

standard. The AUC values were applied for ordinary least squares (OLS) linear regression and for computing the important parameters (kinetic rate constant, half-life time) a programmed excel (Visual Basic for Applications) was utilized. The data are expressed as means of duplicate determinations, and the standard deviations were within 10% of the given values. The calculation of the kinetic rate constant for the degradation and corrected GSH-reactivity is the following. Reaction half-life for pseudo-first order reactions is $t_{1/2}$%=%ln2/k, where k is the reaction rate. In the case of competing reactions (reaction with GSH and degradation), the effective rate for the consumption of the starting compound is k_{eff}%=%k_{deg} + k_{GSH}. When measuring half-lives experimentally, the $t_{1/2(eff)}$%=%ln2/(k_{eff})%=%ln2/(k_{deg} + k_{GSH}). In our case, the corrected k_{deg} and k_{eff} (regarding to blank and GSH containing samples, respectively) can be calculated by linear regression of the data points of the kinetic measurements. The corrected k_{GSH} is calculated by $k_{eff} – k_{deg}$, and finally half-life time is determined using the equation $t_{1/2(GSH)} = \ln2/k_{GSH}$.

LC-MS measurements of antibody conjugates

The molecular weights of the conjugates produced with BUPY (**17**) were identified using a Triple TOF 5600+ hybrid Quadrupole-TOF LC/MS/MS system (Sciex, Singapore, Woodlands) equipped with a DuoSpray IonSource coupled with a Shimadzu Prominence LC20 UFLC (Shimadzu, Japan) system consisting of binary pump, an autosampler and a thermostated column compartment. Data acquisition and processing were performed using Analyst TF software version 1.7.1 (AB Sciex Instruments, CA, USA). Chromatographic separation was achieved on PLRP-S, 1000 Å, 8 µM, 150 mM × 2.1 mM column (Agilent, UK). The separation was achieved using mobile phase A (5% MeCN in 0.1% formic acid) and B (95% MeCN, 5% water 0.1% formic acid) using a gradient elution (Table S1).

The samples were buffer exchanged into ultrapure water and the concentration was set to 5 µM. Before MS measurement, 30 µL of the trastuzumab samples were deglycosylated with 1 µL PNGase F (Glycerol Free) (New England Biolabs Gmbh, P0705), at 37 °C overnight.

Table S1 | Gradient for LC-MS elution.

**Time (min)**	**%A (H_2O 0.1% Fa)**	**%B (MeCN 0.1% FA)**
0	85	15
2	85	15
3	68	32

4	68	32
14	50	50
18	5	95
20	5	95
22	85	15
25	85	15

Flow rate was set to 0.5 ml/min. The column temperature was 60 °C and the injection volume was 10 µl. N_2 was used as the nebulizer gas (GS1), heater gas (GS2), and curtain gas with the optimum values set at 40, 45 and 40 (arbitrary units), respectively. Data were acquired in positive ESI mode in the mass range of m/z=500 to 5000, with 1 s accumulation time. The source temperature was 400°C and the spray voltage was set to 5000 V. Declustering potential value was set to 80 V. PeakView™ V.2.2 (version 2.2, Sciex, Redwood City, CA, USA) was used for deconvoluting the raw electrospray data to obtain the neutral molecular masses.

Biological measurements

Cell lines and culture conditions

MDA-MB-231 control human breast adenocarcinoma, SKOV-3 (HER2+) human ovarian adenocarcinoma cell lines were cultured in sterile culture flasks at 37 °C in a humidified atmosphere with 5% CO2 in an incubator. The cells were cultured in RPMI-1640 (Biosera, Cholet, France), supplemented with 10% FBS (Fetal Bovine Serum South American; Biosera, Cholet, France), Penicillin/Streptomycin Solution 100x (Biosera, Cholet, France). All work with the cell lines were performed in a laminar flow biosafety cabinet.

Flow cytometry

Samples were analysed using an Attune NxT flow cytometer (ThermoFisher Scientific). Trastuzumab fluorescence (BL1-H) was measured with 488 nm excitation and 530/30 nm emission. Analysis was performed using Attune NxT 3.1.2 software. Preparing labelled cells with T-BUPY for FACS MDA-MB-231 control breast and SK-OV-3 (HER2+) ovarian carcinoma cells were harvested with acutase and centrifuged at 300 g for 5 min. One million cells per sample and three replicates per cell line were prepared. The cells were washed in phosphate buffered saline and the supernatant was washed off the cells after each washing step. After the washes, 100 µL of 3% bovine serum albumin (BSA) was added to the cells per tubes and incubated for 30 minutes to block non-specific bindings. After blocking, 100 µL of primary antibody (T-BUPY) (1:1000) was added to the tubes and incubated for 45 minutes at room

temperature. The cells were washed three times with PBS, 100 μL of secondary antibody [Fluorescein (FITC) AffiniPure Goat Anti-Human IgG (Jackson ImmunoResearch Laboratories, 109-095-003, Lot. 147561)] (1:200) was added and incubated in the dark for 45 minutes. After the incubation time, cells were washed three times with PBS, then resuspended in 300 μL PBS per sample.

Preparing labelled cells with T-BUPY for FACS

MDA-MB-231 (low HER2) breast and SKOV-3 (high HER2) ovarian carcinoma cells were harvested with acutase and centrifuged at 300 g for 5 min. One million cells per sample and three replicates per cell line were prepared. The cells were washed in phosphate buffered saline and the supernatant was washed off the cells after each washing step. After the washes, 100 μL of 3% bovine serum albumin (BSA) was added to the cells per tubes and incubated for 30 minutes to block non-specific bindings. After blocking, 100 μL of primary antibody (T-BUPY) (1:1000) was added to the tubes and incubated for 45 minutes at room temperature. The cells were washed three times with PBS, 100 μL of secondary antibody (Fluorescein (FITC) AffiniPure Goat Anti-Human IgG) (1:200) was added and incubated in the dark for 45 min. After the incubation time, cells were washed three times with PBS, then resuspended in 300 μL PBS per sample.

Immunocytochemistry

Glass coverslips were sterilized in 90% ethanol, and after drying they were placed in 6 well cell culture plates (Starstedt, Nümbrech, Germany). 100 µl fibronectin (Sigma, St. Louis, Missouri, USA) per well was added and incubated for 30 minutes in 37 °C termostate. Cells were suspended using trypsin-EDTA (Biosera, Cholet, France) and washed with PBS (Dulbecco's Phosphate Buffered Saline, Biosera, Cholet, France). Approximately 200.000 cells were placed in each well in 1ml of culture medium. After 24 hours of incubation at 37 °C, the cells were washed 3 times with PBS (Biosera, Cholet, France) and then fixed in 4% paraformaldehyde (Thermo Scientific, Waltham, Massachusetts, USA) for 10 minutes. After three PBS washes, we blocked the non-specific protein binding sites with 30 minutes of BSA (Sigma, St. Louis, Missouri, USA) incubation. The cells were incubated with the dye-conjugated primary antibody in 20 µg/mL concentration for 1 hour. After three PBS washes the samples were treated with Hoechst (Sigma, St. Louis, Missouri, USA) for nucleus staining and covered by ProLong Antifade Mountant (Thermo Scientific, Waltham, Massachusetts, USA). The samples were investigated with a Zeiss LSM 710 confocal microscope (Zeiss, Jena, Germany).

MTT assay

Cell viability was determined by MTT assay (3-(4,5-dimethylthiazol-2-yl)-2,5-diphenyl-tetrazolium bromide) which was obtained from Duchefa Biochemie (Haarlem, The Netherlands). After standard harvesting of the cells by trypsin-EDTA (Lonza) and phosphate-buffered saline (PBS, Lonza), 5 × 103 from MDA-MB-231 low HER2 breast (control) and 6×103 cells per well from SKOV-3, HER2 positive ovarium carcinoma cell lines were seeded in 5% serum containing growth medium to 96-well plates with flat bottom (Sarstedt), in a 100 µL volume per well, and incubated at 37 °C. After 24 h, cells were treated with various concentrations of compounds (50 µg/mL – 0,2 µg/mL), dissolved in medium (0.5% final, Sigma Aldrich, St. Louis, MO, USA) and serum free medium (serum final 2.5%) and incubated for 72 h under standard conditions. The control wells were treated with serum free medium. Afterward, the MTT assay was performed, in order to determine cell viability, by adding 20 µL of MTT solution (5 mg/mL in PBS, 0.5 mg/mL final) to each well and after 2 h of incubation at 37 °C, the supernatant was removed. The precipitated purple formazan crystals were dissolved in 100 µL of a 1:1 solution of dimethyl sulfoxide (DMSO; Sigma Aldrich) – 96% Ethanol (Molar Chemicals Kft., Hungary) and the absorbance was measured after 15 min. at λ = 570 nm by using The Spark microplate reader (TECAN, Morgen Hill, CA, USA). Average background absorbance (DMSO–Ethanol) was subtracted from absorbance values of control and treated wells, and cell viability was determined relative to untreated (control) wells where cell viability was arbitrarily set to 100%. Absorbance values of treated samples were normalized versus untreated control samples and interpolated by Dose-response curve non-linear 4-parameter (variable slope) regression analysis with GraphPad Prism 6 software (GraphPad, La Jolla, San Diego, CA, USA) to generate sigmoidal dose-response curves from which the half maximal inhibitory concentration (IC50) values of the compounds were calculated. The experiments were done in triplicate and each experiment was repeated twice.

Synthetic procedures

3,5-;2,3-;2,5-Dibromo-1-methylpyrazin-1-ium-trifluoromethanesulfonate ***(14a, 14b, 14c)***

2,3-; 2,5- or 2,6-dibromopyrazine (**13a,b,c**) (0.44 mmol) was dissolved in acetonitrile (5 mL) and added methyl trifluoromethanesulfonate (48 µL, 0.44 mmol, 1 equiv.). The reaction mixture

was stirred in the dark at 25 °C for 30 min. After this time, the reaction mixture was concentrated *in vacuo*. The crude reaction mixture was then dissolved three times in acetonitrile (3x50 mL) and once in dichloromethane (50 mL) and concentrated again each time. Diisopropyl ether was added to the crude residue and cooled to 0 °C. The crystals are filtered and washed with diethyl ether.

Product **14a** is a brown crystalline solid (35.0 mg, 18%); m.p.: 70-72 °C (Et_2O); ^{1}H NMR (300 MHz, DMSO-d_6) δ 7.79 (d, J = 4.03 Hz, 1H, ArH), 7.18 (d, J = 4.03 Hz, 1H, ArH), 3.51 (s, 3H, NCH_3) ppm; ^{13}C NMR (75 MHz, DMSO-d_6) 132.8 (=CH), 122.3 (=CH), 108.1 (C=), 92.1 (C=), 35.5 (NCH_3) ppm; HRMS: $[M]^+$ found: 250.8811, calcd: 250.8813.

Product **14b** is a brown crystalline solid (83.6 mg, 45%); m.p.: 119-121 °C (Et_2O); ^{1}H NMR (300 MHz, DMSO-d_6) δ 8.11 (s, 1H, ArH), 7.86 (s, 1H, ArH), 3.43 (s, 3H, NCH_3) ppm; ^{13}C NMR (DMSO-*d*6, 75 MHz) 147.5 (=CH), 133.2 (=CH), 113.0 (C=), 90.5 (C=), 36.9 (NCH_3) ppm; HRMS: $[M]^+$ found: 250.8811, calcd: 250.8813.

Product **14c** is a brown crystalline solid (64.5 mg, 34%); m.p.: 189-191 °C (Et_2O); ^{1}H NMR (300 MHz, MeCN-d_3) δ 9.07(s, 2H, ArH), 4.36 (s, 3H, NCH_3) ppm; ^{13}C NMR (125 MHz, MeCN-d_3) δ 142.4 (=CH), 140.1 (C=), 49.0 (NCH_3) ppm; HRMS: $[M]^+$ found: 250.8817, calcd: 250.8813.

*3,5-Dibromo-1-N-but-3-yn-1-yl-pyrazin-1-ium trifluoromethanesulfonate (**17**)*

But-3-yn-1-ol (**16**) (0.15 mL, 2.0 mmol, 4.0 equiv.), sodium carbonate (0.13 g, 1.30 mol, 2.6 equiv.) was dissolved in dichloromethane (40 mL). While the components were added to the solution, the mixture was continuously purged with nitrogen. The reaction mixture was stirred and cooled to -50 °C. After the mixture reached the appropriate temperature, then trifluoromethanesulfonic anhydride (0.42 mL, 2.5 mmol, 5.0 equiv.) was added dropwise. The reaction mixture was stirred in dark at 25 °C for 3 hours and evaporated under reduced pressure. 2,6-dibromopyrazine (**13a**) (0.12 g, 0.5 mmol) was dissolved in acetonitrile (80 mL) and the evaporated alkylating agent was added to this solution, then stirred for 1 h in dark. Purification of the crude residue by flash column chromatography yielded product **17** (152.0 mg, 69%) as a yellow crystalline solid; m.p.: 195-196 °C (Et_2O); ^{1}H NMR (500 MHz, MeCN-d_3) δ 9.15 (s, 2H, ArH), 4.72 (t, J = 6.71 Hz, 2H, NCH_2), 3.03-2.98 (m, 2H, CH_2), 2.59 (t, J = 3.05 Hz, 1H,

HC≡) ppm; ^{13}C NMR (MeCN-d_3, 125 MHz) δ 142.8 (=CH), 139.1 (C=), 77.3 (C=), 74.5 (≡CH), 60.8 (NCH_2), 20.2 (CH_2) ppm; HRMS: $[M]^+$ found: 288.8966, calcd: 288.8970.

*(14S,16S,32S,33S,2R,4S,10E,12E,14R)-86-chloro-14-hydroxy-85,14-dimethoxy-33,2,7,10-tetramethyl-12,6-dioxo-7-aza-1(6,4)-oxazinana-3(2,3)-oxirana-8(1,3)-benzenacyclotetradecaphane-10,12-dien-4-yl N-(3-((1-((4-((3-azidopropyl)carbamoyl)cyclohexyl)methyl)-2,5-dioxopyrrolidin-3-yl)thio)propanoyl)-N-methyl-L-alaninate (**18**)*

In a round bottom flask 10 mg [(1*S*,2*R*,3*S*,5*S*,6*S*,16*E*,18*E*,20*R*,21*S*)-11-chloro-21-hydroxy-12,20-dimethoxy-2,5,9,16-tetramethyl-8,23-dioxo-4,24-dioxa-9,22-diazatetracyclo[19.3.1.110,14.03,5]hexacosa-10,12,14(26),16,18-pentaen-6-yl] (2*S*)-2-[methyl(3-sulfanylpropanoyl)amino]propanoate (DM1, mertansine) (0,014 mmol) was dissolved in 10 ml acetonitrile. To this solution 10.3 mg succinimidyl 4-[*N*-maleimidomethyl]cyclohexane-1-carboxylate (SMCC) (0.013 mmol) and 0.24 µL DIPEA (4.4 mg, 0.0014 mmol, 0.1 eq.) was added and stirred for 30 minutes. In the next step 1.7 mg 3-azidopropan-1-amine (0,017 mmol, 1.25 eq.) and 2.4 µL DIPEA (44 mg, 0.014 mmol, 1 eq) was added and stirred for overnight at room temperature in dark. The next morning the mixture was evaporated and purified by preparative HPLC. The purification yielded product **18** (10.8 mg, 75%) as a white crystalline solid. ^{1}H NMR assignation of **18** N_3-SMCC-DM1 was made based on literature reference.[S1]

^{1}H NMR (500 MHz, $CDCl_3$) δ 6.85 (d, J = 8.7 Hz, 1H, ArH, ***20***), 6.73 – 6.69 (m, 1H, ArH, ***2***), 6.69 - 6.66 (m, 1H, HC=, ***6***), 6.45 (dd, J = 15.1, 11.3 Hz, 1H, HC=, ***7***), 6.24 (s, 1H, HC=, ***5***), 5.76 – 5.70 (m, 1H, NH, ***36***), 5.67 (dd, J = 15.4, 9.2 Hz, 1H, CH, ***21***), 5.41 - 5.32 (m, 1H, CH, ***13***), 4.78-4.68 (m, 1H, CH, ***8***), 4.37 – 4.28 (m, 1H, CH, ***17***), 4.01 (s, 3H, OCH_3, ***1***), 3.71 – 3.54 (m, 2H, CH2, ***3***), 3.54 – 3.49 (m, 1H, ***27***), 3.41 – 3.39 (m, 2H, CH_2, ***12***), 3.37 (s, 3H, NCH_3, ***19***), 3.36 – 3.32 (m, 2H, CH_2, ***37***), 3.30 - 3.24 (m, 1H, C*H*H, ***24***), 3.22 (s, 3H, OCH_3, ***9***), 3.17 – 3.10 (m, 2H, CH_2, ***25***), 3.10 – 3-05 (m, 1H, NH, ***11***), 3.05 – 2.98 (m, 2H, ***29***), 2.92 – 2.80 (m, 4H, NCH_3, ***23***, CH, ***14***), 2.73 – 2.54 (m, 2H, CH_2, CH_2, ***18***), 2.45 – 2.33 (m, 1H, CH*H*, ***24***), 2.27 – 2.16 (m, 1H, CH,***16***), 2.08 – 1.95 (m, 1H, CH, ***33***), 1.93 – 1.84 (m, 2H, CH_2 eq, ***32***, ***34***), 1.85 – 1.77 (m, 2H, CH_2, ***38***), 1.75-1.70 (m, 2H, CH_2 eq, ***31***, ***35***), 1.67 (s, 3H, CH_3, ***4***), 1.52 – 1.41 (m, 2H, CH_2, ***39***), 1.37 – 1.26 (m, 11H, CH_3, ***22***, ***26***, CH_2 ax, 31, 35,

CH_2, ***28***, CH, **30**), 1.08 – 0.93 (m, 2H, CH_2 ax, ***32***, ***34***), 0.83 (s, 3H, CH_3, ***15***) ppm. ^{13}C NMR (MeCN-d_3, 125 MHz) δ 178.3, 176.6, 176.2, 172.1, 171.9, 169.8, 157.2, 157.0, 152.6, 142.9, 142.6, 140.6, 134.4, 129.1, 126.2, 123.4, 114.92, 114.85, 89.2, 81.9, 79.1, 74.9, 68.0, 61.2, 57.5, 57.1, 53.4, 53.3, 49.9, 47.0, 45.7, 45.4, 40.7, 39.4, 37.1, 36.9, 36.8, 36.7, 36.6, 36.0, 35.9, 35.1, 34.9, 33.3, 31.1, 31.0, 30.7, 29.8, 29.7, 27.8, 27.7, 15.6, 14.8, 13.8, 12.5 ppm; HRMS: [M+H]+ found: 1057.4438, calcd:1057.4471.

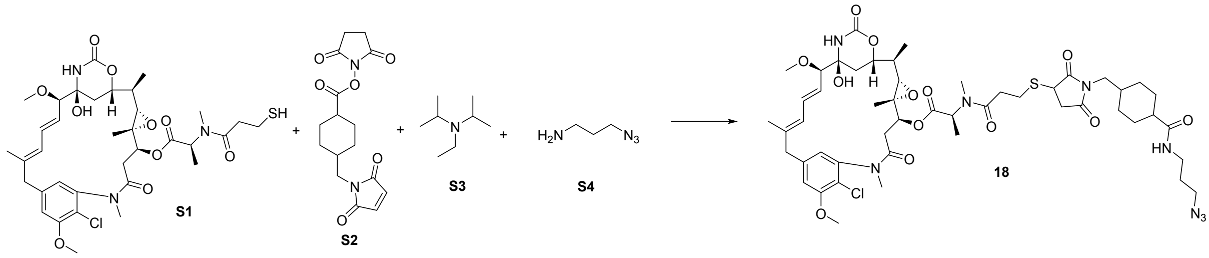

Scheme S1. Synthesis of azido-SMCC-DM1 (**18**).

Results of HPLC-MS kinetic measurements

Table S2: *GSH-reactivity and aqueous stability assay results of the covalent probes (**14a-c**)*

Entry	Compound	GSH-stability and aqueous stability assay				
		k_{deg} [h^{-1}]	k_{eff} [h^{-1}]	k_{GSH} [h^{-1}]	$t_{1/2(stability)}$ [h]	$t_{1/2(GSH)}$ [h]
1	**14a**	N/D	N/D	N/D	<1	N/D
2	**14b**	N/D	N/D	N/D	<1	N/D
3	**14c**	N/D	N/D	N/D	<1	<0.05
4	**15**	0.127	N/D	N/D	5.5	<0.05

N/D: not determined (due to high instability and/or hyperreactivity). The reactions faster than the minimal time window necessary to obtain LC-MS spectra are reported here with a half-life < 0.05 h, due to the minimal running time was 3 min.

Trastuzumab Fab and mAb conjugation methods

<u>Trastuzumab Fab generation</u>

Fab was generated from trastuzumab *via* enzymatic digestion following a literature procedure detailed in: F. Thoreau et al. *ACS Cent. Sci.* **2023**, 9, 3, 476–487.

<u>Fab_{HER2} rebridging with BUPY</u>

To 20 µL 20 µM Fab_{HER2} (0.0004 µmol) solution in PBS buffer (pH= 6.0, 7.0 or 8.0) 50 equiv. of TCEP (0.02 µmol, 10 µL 2 mM TCEP stock solution in water) was added. The reaction mixture was incubated for 90 minutes at 37 °C under constant agitation (300 rpm). After this time, TCEP was removed using a ZebaSpin 7 kDa MWCO desalting column into pH=6 50 mM PBS buffer. To the reaction was added BUPY (0.2 µL, 10 Mm, 5 eq.) and was incubated for 90 minutes at 37 °C under constant agitation (300 rpm). After this time, the protein was purified from the small molecular reactants with VivaSpin 500 10 kDa MWCO membrane filter twice and ZebaSpin 7 kDa MWCO columns once. The conjugate was analyzed with UHPLC-MS, and non-reducing SDS-PAGE. The DOL was calculated from the MS spectrum.

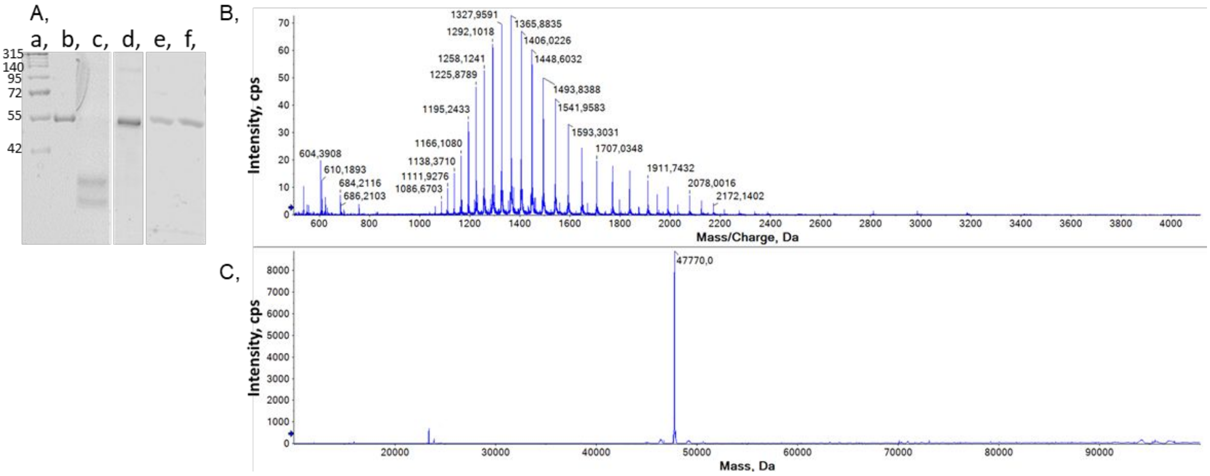

Figure S1. SDS-PAGE of Fab_{HER2} rebridging at pH = 6,7,8 and MS spectra of Fab_{HER2}-BUPY conjugate. A, a, Protein marker. b, Native Fab_{HER2}. c, Reduced Fab_{HER2}. d, Rebridging of Fab_{HER2} with BUPY (**17**) at pH=8. e, Rebridging of Fab_{HER2} with BUPY (**17**) at pH=7. f, Rebridging of Fab_{HER2} with BUPY (**17**) at pH=6. B, Raw mass spectrum, C, Deconvoluted mass spectrum; expected: 47771 Da, observed: 47770 Da.

<u>Examining the selectivity of BUPY</u>

To 100 µL 5 µM Fab_{HER2} solution (0.0005 µmol) in pH=6 PBS buffer, 2.0 µL 10 mM BUPY stock solution in water (40 equiv., 0.02 µmol) was added and incubated for 2 hours at room temperature. After this time, buffer exchange was done two times with Vivaspin500^{TM} 10 kDa MWCO membrane filter and once with Zeba™ Spin 7K MWCO, 0.5 ml. The sample was examined by MS measurements and SDS-PAGE.

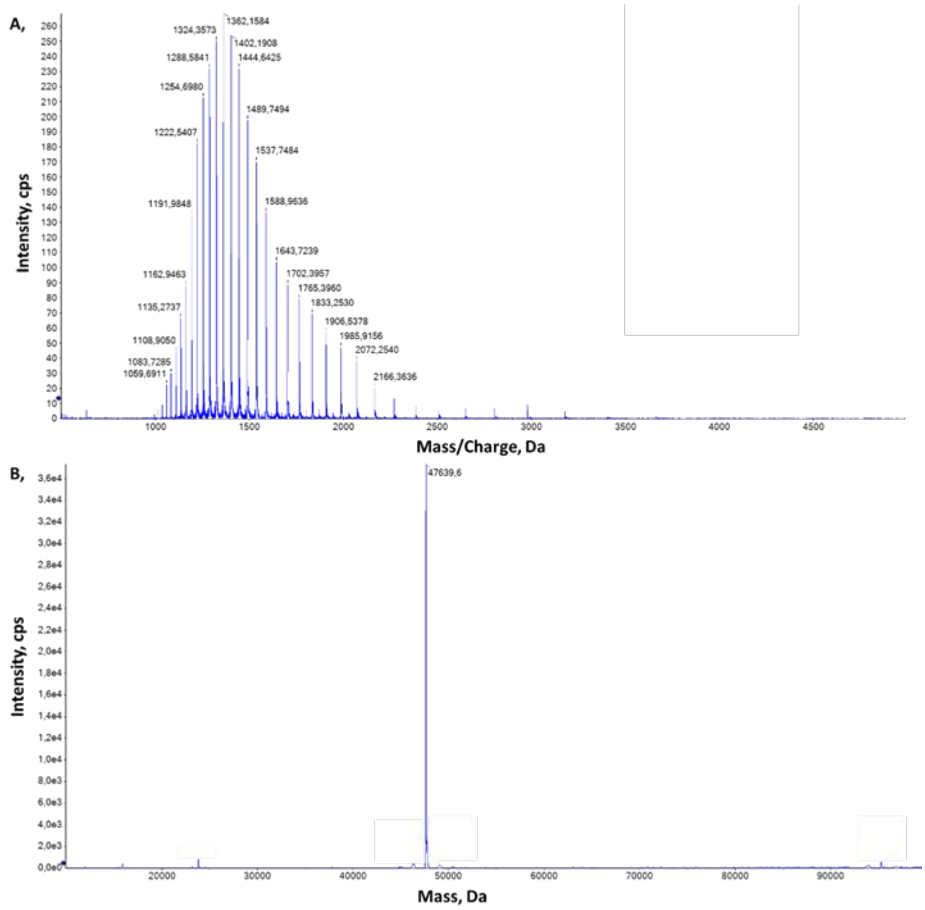

Figure S2. MS spectra and SDS-PAGE analysis of selectivity examination with Fab_{HER2} and BUPY. A, Raw mass spectrum, B, Deconvoluted mass spectrum; expected: 47639 Da, observed: 47639 Da.

Fab_{HER2}-BUPY click reaction with DM1-azide

To 85 µL 19.3 µM Fab_{HER2}-BUPY solution (0.0016 µmol) in PBS was added 51 µL PBS and 13.6 µL DMF. 0.022 µmol azide (DM1-azide, final concentration 146 µM), $CuSO_4 \cdot 5H_2O$ (final concentration of 244 µM), THPTA (final concentration of 1.22 mM) and sodium ascorbate (final concentration of 1.83 mM). The reaction mixture was incubated at 37 °C overnight. After this time, buffer exchange was done two times with Vivaspin500™ 10 kDa MWCO membrane filter and once with Zeba™ Spin 7K MWCO, 0.5 ml. The conjugate was investigated with UV/Vis absorbance measurement, UPLC-MS and non-reducing SDS-PAGE. The DOL was calculated from MS: DOL=1. The DOL was determined also from UV absorbance at 252 and 280 nm. The molar extinction coefficients were: λ_{252}^{Fab} = 20200 $cm^{-1}M^{-1}$, λ_{280}^{Fab} = 68400 $cm^{-1}M^{-1}$, λ_{252}^{DM1} = 28480 $cm^{-1}M^{-1}$, λ_{280}^{DM1} = 17720 $cm^{-1}M^{-1}$. The calculation using the Lambert-Beer equation gives 1.1 DM1-antibody ratio on average.

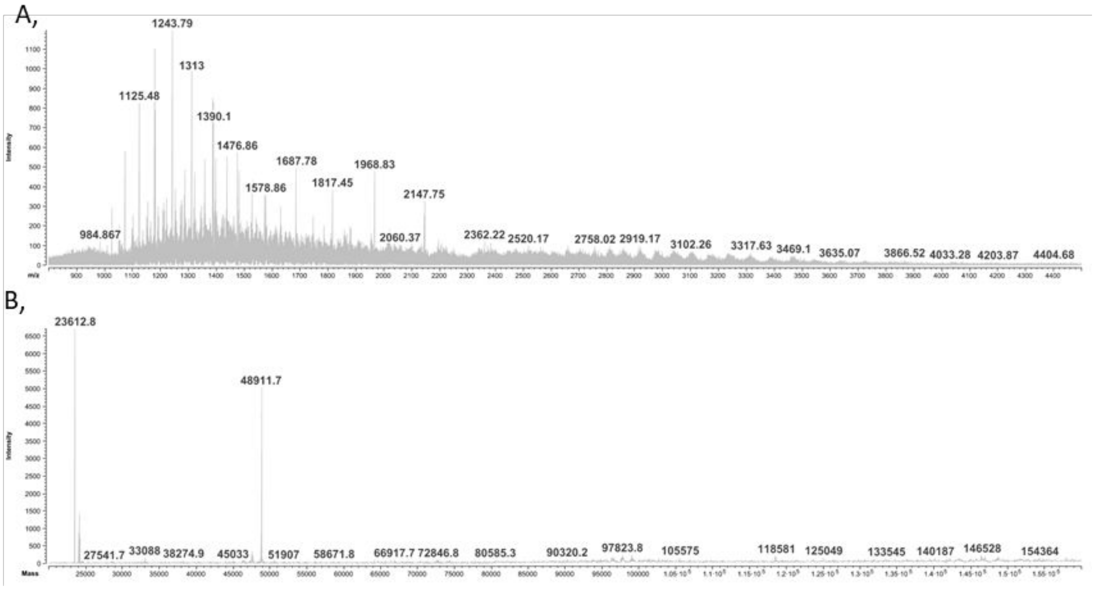

Figure S3. MS spectra of Fab_{HER2}-BUPY conjugate. A, Raw mass spectrum, B, Deconvoluted mass spectrum; expected (Fab_{HER2}-BUPY-DM1+2 MeCN): 48 909 Da, observed: 48911 Da.

<u>Trastuzumab rebridging with BUPY</u>

In a microcentrifuge tube 78 µL 10 µM trastuzumab (0.0008 µmol) solution in pH=6 PBS buffer was treated with 20 equiv. (0.016 µmol, 4 µL 4 mM TCEP stock solution in water). After 90 minutes incubation at 37 °C and under constant agitation (300 rpm) the TCEP was removed and buffer exchanged to pH=6 PBS buffer with ZebaSipn 7 kDa MWCO column. To the solution 0.8 µL 10 mM BUPY stock solution (0.008 µmol, 10 equiv.) was added. The mixture was incubated at 37 °C under constant agitation (300 rpm) for 1 hour. After this time, buffer exchange was done two times with Vivaspin500™ 10 kDa MWCO membrane filter and once with Zeba™ Spin 7K MWCO, 0.5 ml. The conjugate was investigated with UV/Vis absorbance measurement and non-reducing SDS-PAGE. The DOL was calculated from the absorbance and the MS spectra. Both gave a 4:1 BUPY to antibody ratio.

During optimization the same procedures were performed with 5, 20 or 40 eq. of BUP and 5 or 20 µM trastuzumab and incubated over 6 hours with samples taken every hour for analysis.

The distribution of species by SDS-PAGE densitometry was found to be: 47±1.1% fully rebridged antibody, 3±2.4% rebridged HH, 46±2.2% rebridged half antibody, 2±1.0% H and 1±1.1% L.

Conjugation efficiency 97%±2%.

DOL calculation

$$A_{252\,nm} = \varepsilon_{BuPy,252\,nm} \cdot l \cdot c_{\beta C} + \varepsilon_{T,252\,nm} \cdot l \cdot c_T$$

$$A_{330\,nm} = \varepsilon_{BuPy,330\,nm} \cdot l \cdot c_{\beta C} + \varepsilon_{T,330\,nm} \cdot l \cdot c_T$$

Combined together the two equations with CF factor what can be determined by the following way: c_{BUPY}/c_T

$$DAR = \frac{\dfrac{A_{330\,nm}}{\varepsilon_{BuPy,330\,nm}}}{\dfrac{A_{252\,nm} - CF \cdot A_{330\,nm}}{\varepsilon_{trastuzumab,252\,nm}}}$$

We calculated average from the 3 parallel absorbance measurement at the different wavelengths. The average absorbance values than were included into the complex equation together with the other constants:

$$DAR = \frac{\dfrac{0.0087}{3828\,M^{-1} \cdot cm^{-1}}}{\dfrac{0.0497 - 0.37 \cdot 0.0087}{81924\,M^{-1} \cdot cm^{-1}}} = 4.0$$

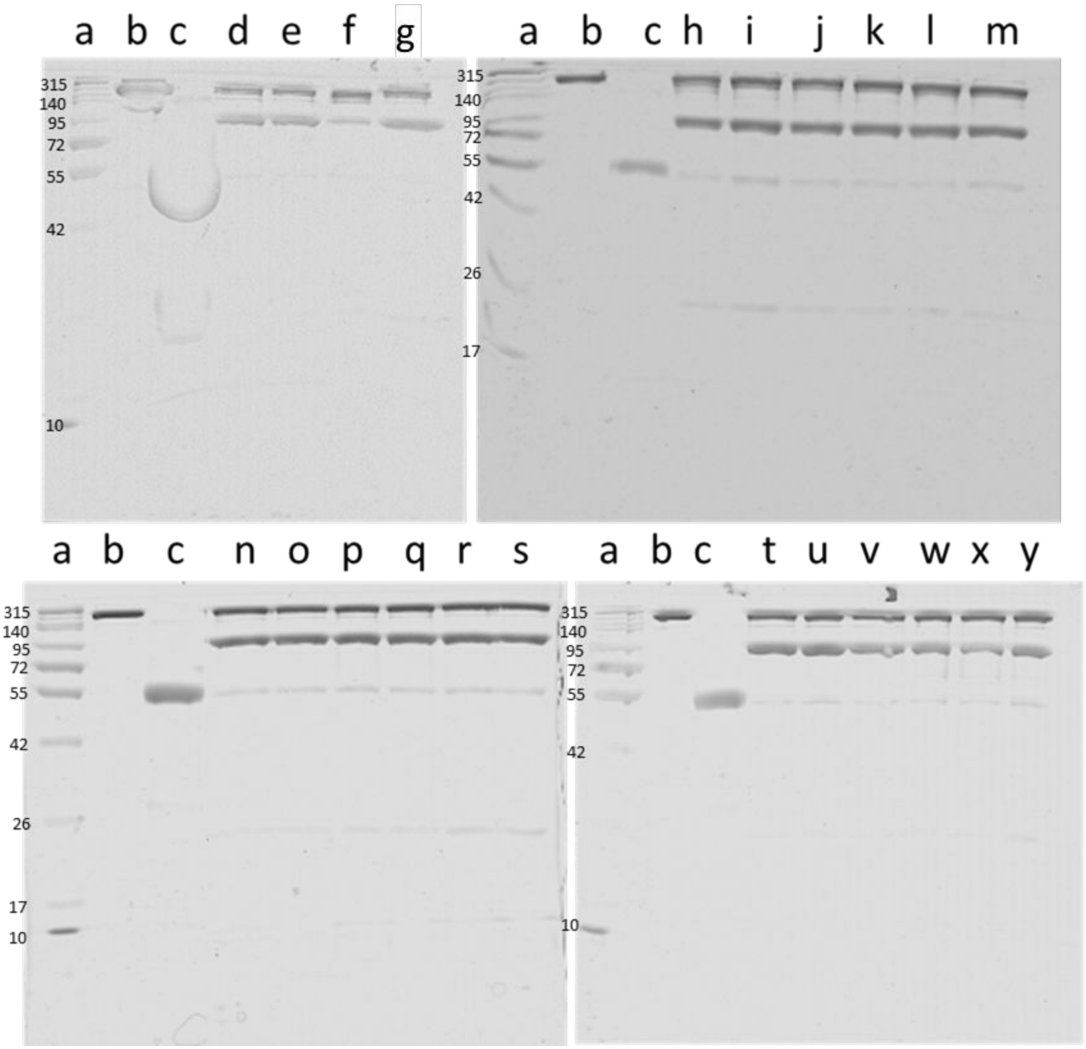

Figure S4. SDS-PAGE analysis of BUPY rebridging optimalization. a, Protein marker. b, Native trastuzumab. c, Reduced trastuzumab. d, Rebridging at pH=6 with 5 eq. BUPY and 5 µM mAb. e, Rebridging at pH=6 with 10 eq. BUPY and 5 µM mAb. f, Rebridging at pH=6 with 20 eq. BUPY and 5 µM mAb. g, Rebridging at pH=6 with 40 eq. BUPY and 5 µM mAb. h, Rebridging at pH=6 with 10 eq. BUPY and 20 µM mAb by 1 h. i, Rebridging at pH=6 with 10 eq. BUPY and 20 µM mAb by 2 h. j, Rebridging at pH=6 with 10 eq. BUPY and 20 µM mAb by 3 h. k, Rebridging at pH=6 with 10 eq. BUPY and 20 µM mAb by 4 h. l, Rebridging at pH=6 with 10 eq. BUPY and 20 µM mAb by 5 h. m, Rebridging at pH=6 with 10 eq. BUPY and 20 µM mAb by 6 h. n, Rebridging at pH=6 with 10 eq. BUPY and 10 µM mAb by 1 h. o, Rebridging at pH=6 with 10 eq. BUPY and 10 µM mAb by 2 h. p, Rebridging at pH=6 with 10 eq. BUPY and 10 µM mAb by 3 h. q, Rebridging at pH=6 with 10 eq. BUPY and 10 µM mAb by 4 h. r, Rebridging at pH=6 with 10 eq. BUPY and 10 µM mAb by 5 h. s, Rebridging at pH=6 with 10 eq. BUPY and 10 µM mAb by 6 h. t, Rebridging at pH=6 with 10 eq. BUPY and 5 µM mAb by 1 h. u, Rebridging at pH=6 with 10 eq. BUPY and 5 µM mAb by 2 h. v, Rebridging at pH=6 with 10 eq. BUPY and 5 µM mAb by 3 h. w, Rebridging at pH=6 with 10 eq. BUPY and 5 µM mAb by 4 h. x, Rebridging at pH=6 with 10 eq. BUPY and 5 µM mAb by 5 h. y, Rebridging at pH=6 with 10 eq. BUPY and 5 µM mAb by 6 h.

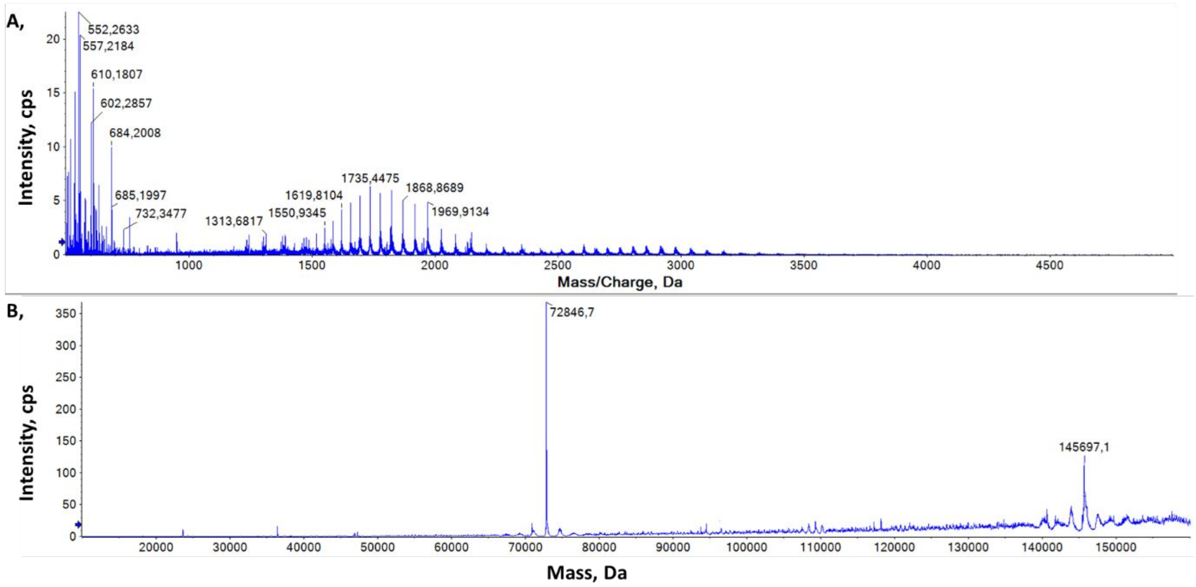

Figure S5 MS spectra of trastuzumab-BUPY conjugate. A, Raw mass spectrum, B, Deconvoluted mass spectrum; expected: 145694, 72847 Da, observed: 145697, 72847 Da.

<u>Click reaction of 5-TAMRA and T-BUPY conjugate</u>

Click reaction of 5-TAMRA and DM1 with T-BUPY conjugate

To 53 µL 118.3 µM T-BUPY solution (0.00627 µmol) in PBS was added 0.0643 µmol azide (DM1-azide, 5-TAMRA-PEG3-azide, final concentration 164 µM) $CuSO_4·5\ H_2O$ (final concentration of 274 µM), THPTA (final concentration of 1.37 mM) and sodium ascorbate (final concentration of 2.06 mM). The reaction mixture was incubated at 37 °C overnight. After this time, buffer exchange was done two times with Vivaspin500™ 10 kDa MWCO membrane filter and once with Zeba™ Spin 7K MWCO, 0.5 ml. The conjugate was investigated with UV/Vis absorbance measurement, UPLC-MS and non-reducing SDS-PAGE.

The DOL was determined from UV absorbance at 252 and 280 nm (Figure S6). The molar extinction coefficients (ε) were: $\varepsilon_{252}^{T} = 68580\ cm^{-1}M^{-1}$, $\varepsilon_{280}^{T} = 225000\ cm^{-1}M^{-1}$, $\varepsilon_{252}^{DM1} = 28480\ cm^{-1}M^{-1}$, $\varepsilon_{280}^{DM1} = 17720\ cm^{-1}M^{-1}$. The calculation using the Lambert-Beer equation gave 4.0:1 DM1 to antibody ratio on average.

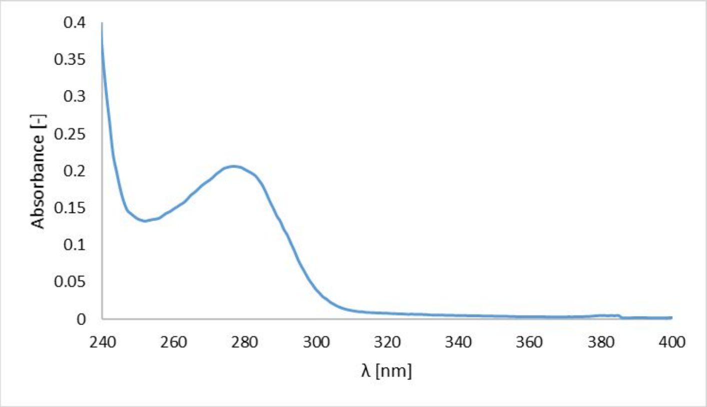

Figure S6. UV spectrum of T-BUPY-M

The FAR (fluorophore antibody ratio) was determined from UV absorbance at 252 and 280 nm (Figure S7). The molar extinction coefficients were: $\varepsilon_{280}^{T} = 225000\ cm^{-1}M^{-1}$, $\varepsilon_{552}^{T} = 0\ cm^{-1}M^{-1}$, $\varepsilon_{280}^{TAMRA} = 9340\ cm^{-1}M^{-1}$, $\varepsilon_{280}^{TAMRA} = 40740\ cm^{-1}M^{-1}$. The calculation using the Lambert-Beer equation gave 4.1:1 TAMRA to antibody ratio on average.

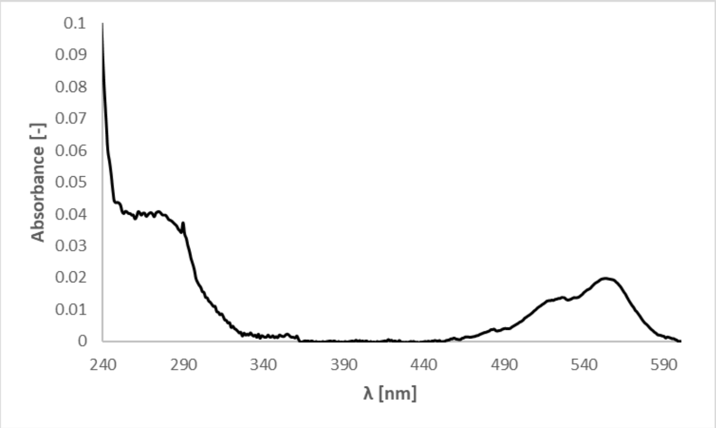

Figure S7. UV spectrum of T-BUPY-TAMRA

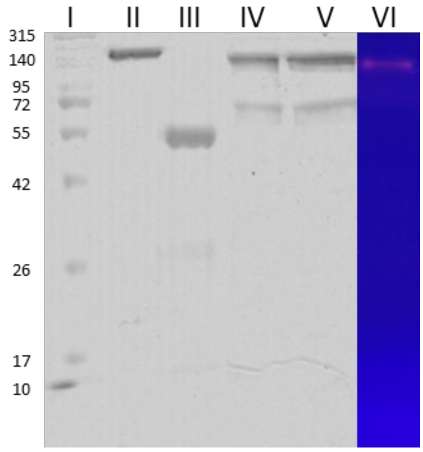

Figure S8. SDS-PAGE analysis of clicked products. I. Protein marker. II. Native trastuzumab. III. Reduced trastuzumab. IV. T-BUPY-TAMRA Coomassie stained. V. T-BUPY-M Coomassie stained. VI. T-BUPY-TAMRA excited by 366 nm light.

Serum stability examination of T-BUPY-TAMRA conjugate

To solution of T-BUPY-TAMRA (17 µL, 8.65 µM) in PBS was added 22,5 µL of reconstituted human plasma (Sigma) and 223 µL of PBS. To this solution was added reduced L-glutathione (0.26 µL, 1 mM) and the mixture incubated at 37 °C for 7 days. Aliquots were removed after 0, 1, 2, 3, 4, 5, 6, 7 days, flash frozen and stored at -20 °C until analysis. SDS-PAGE was followed by in-gel fluorescence and Coomassie Brilliant Blue staining.

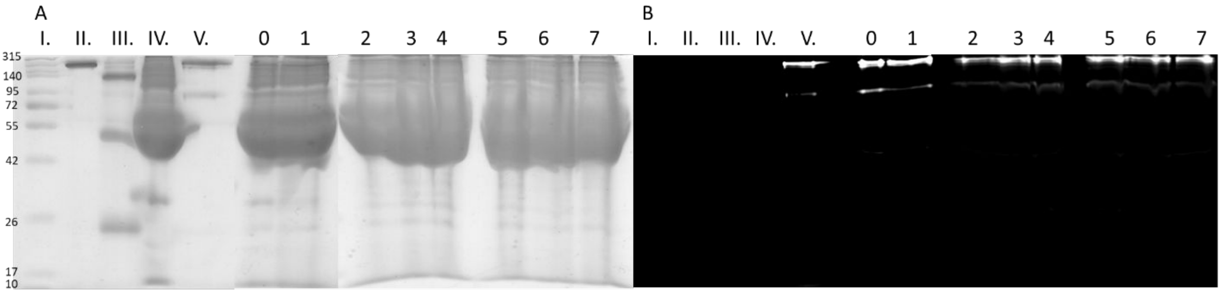

Figure S9. SDS-PAGE analysis of stability. A, Coomassie stained gel, B, fluorescence of gel recorded before staining. I. Protein marker, II. Native trastuzumab, III. Reduced trastuzumab, IV. Bovine serum, V. T-BUPY-TAMRA, The numbers from 0 to 7 refer to the days of T-BUPY-TAMRA incubation in bovine serum.

NMR spectra

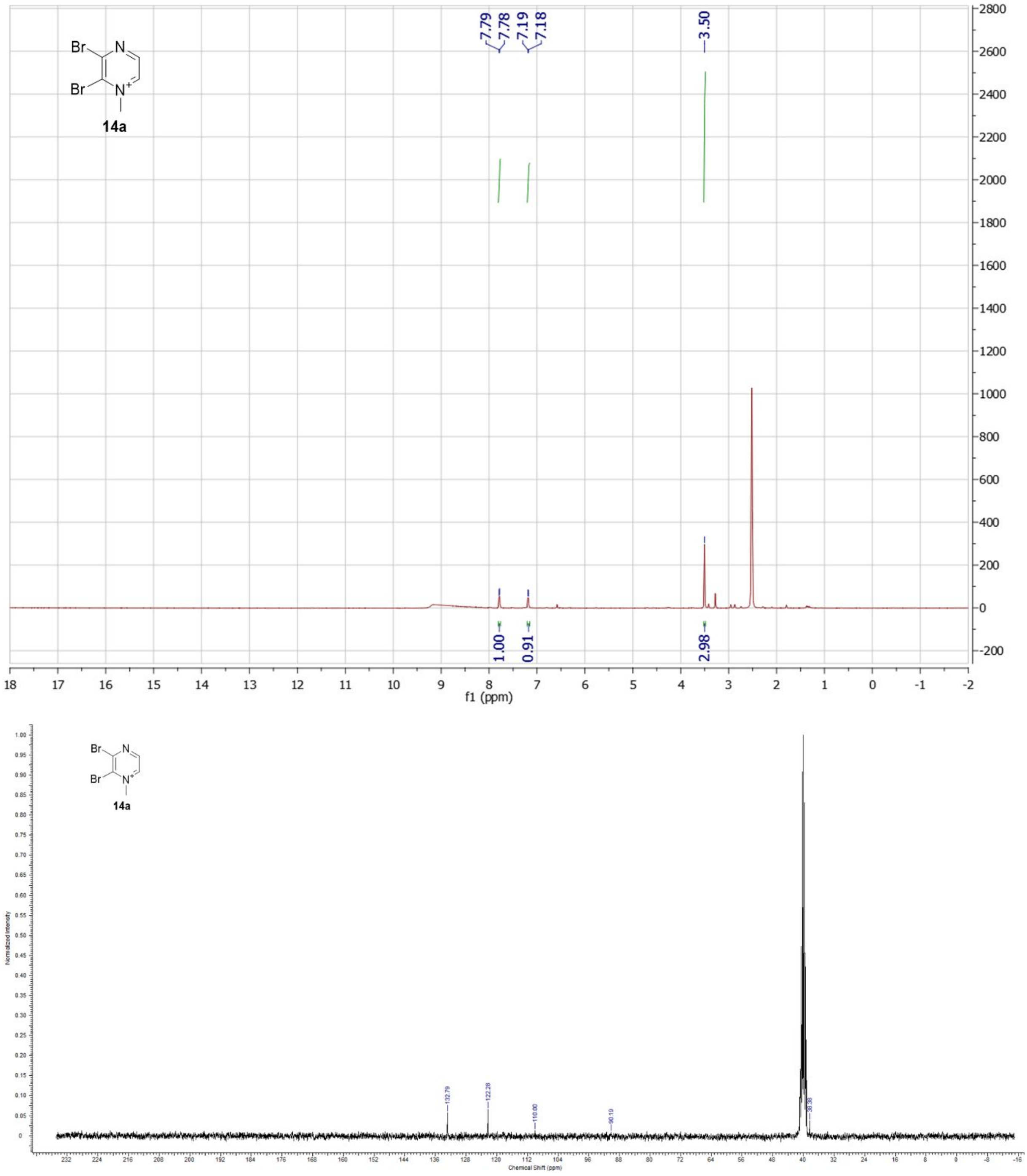

Figure S9. NMR spectra of **14a** pyrazinium trifluoromethanesulfonate

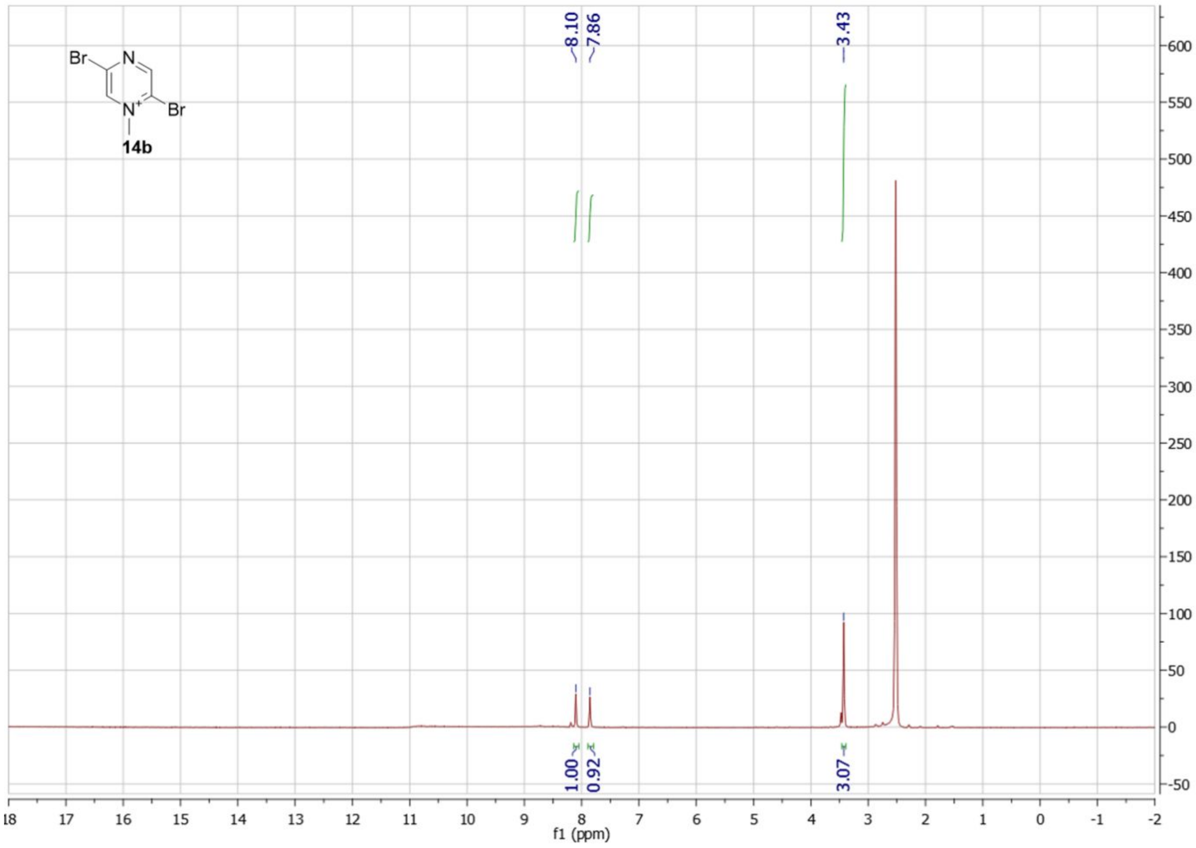

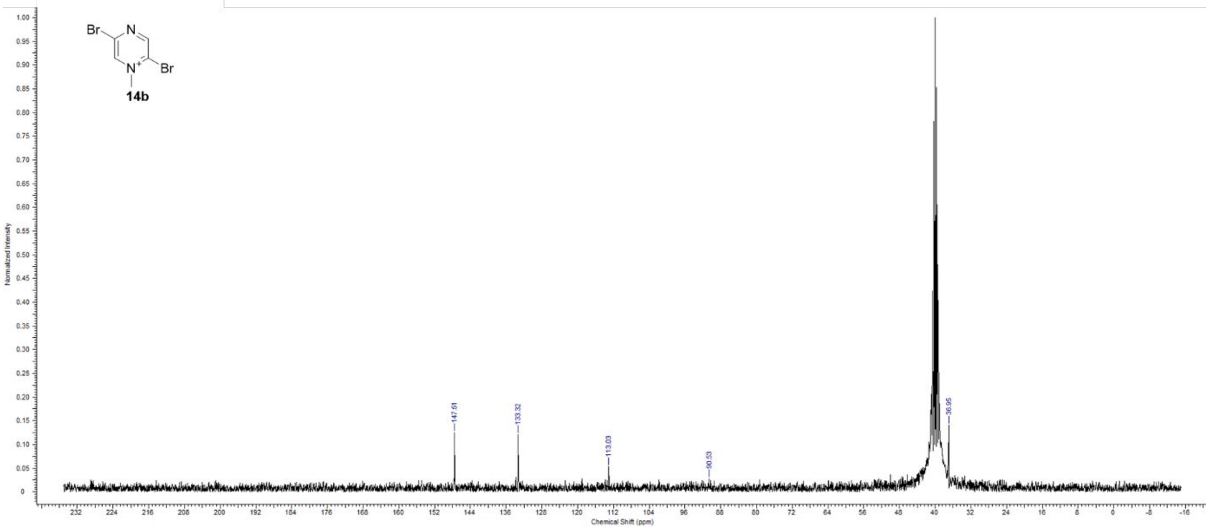

Figure S10. NMR spectra of **14b** pyrazinium trifluoromethanesulfonate

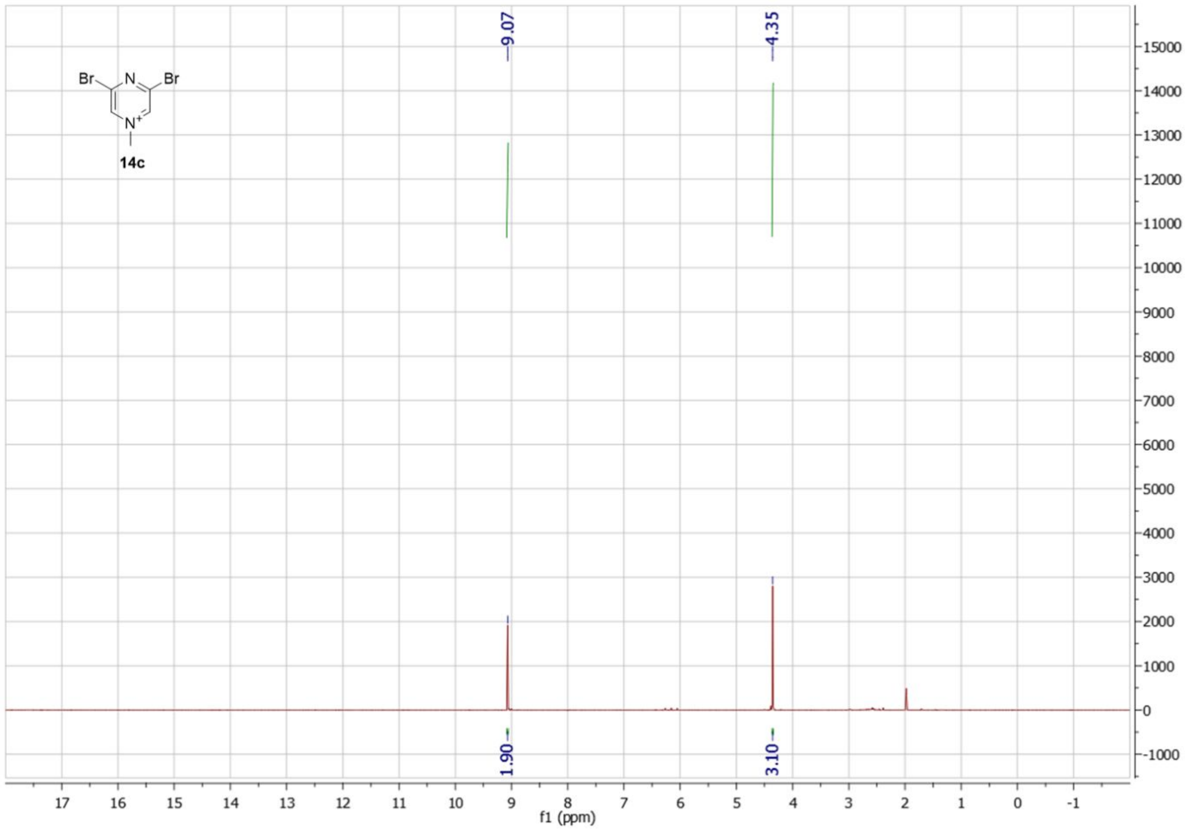

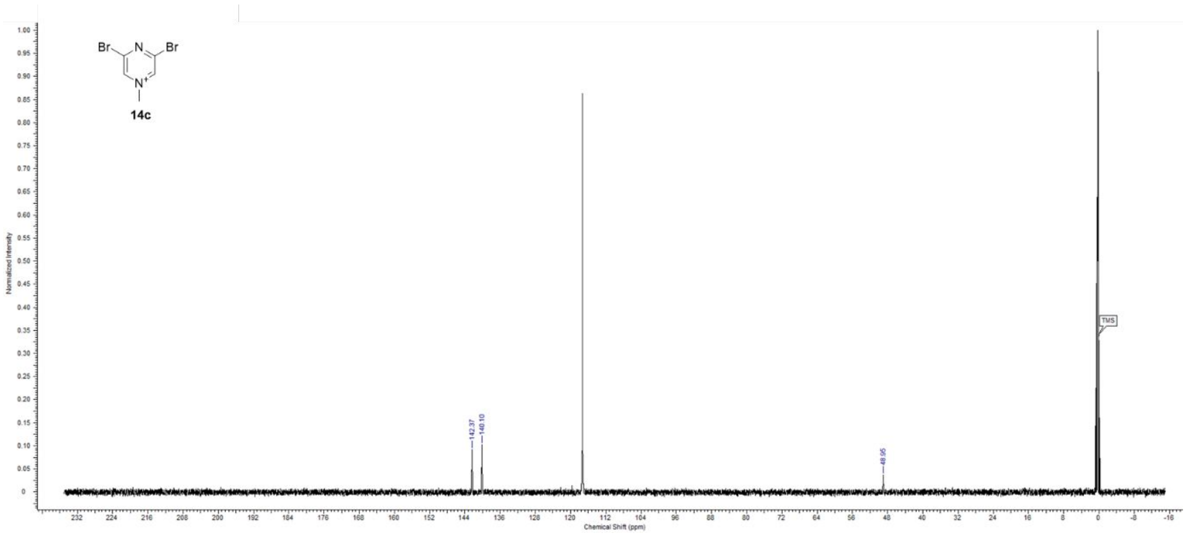

Figure S11. NMR spectra of **14c** pyrazinium trifluoromethanesulfonate

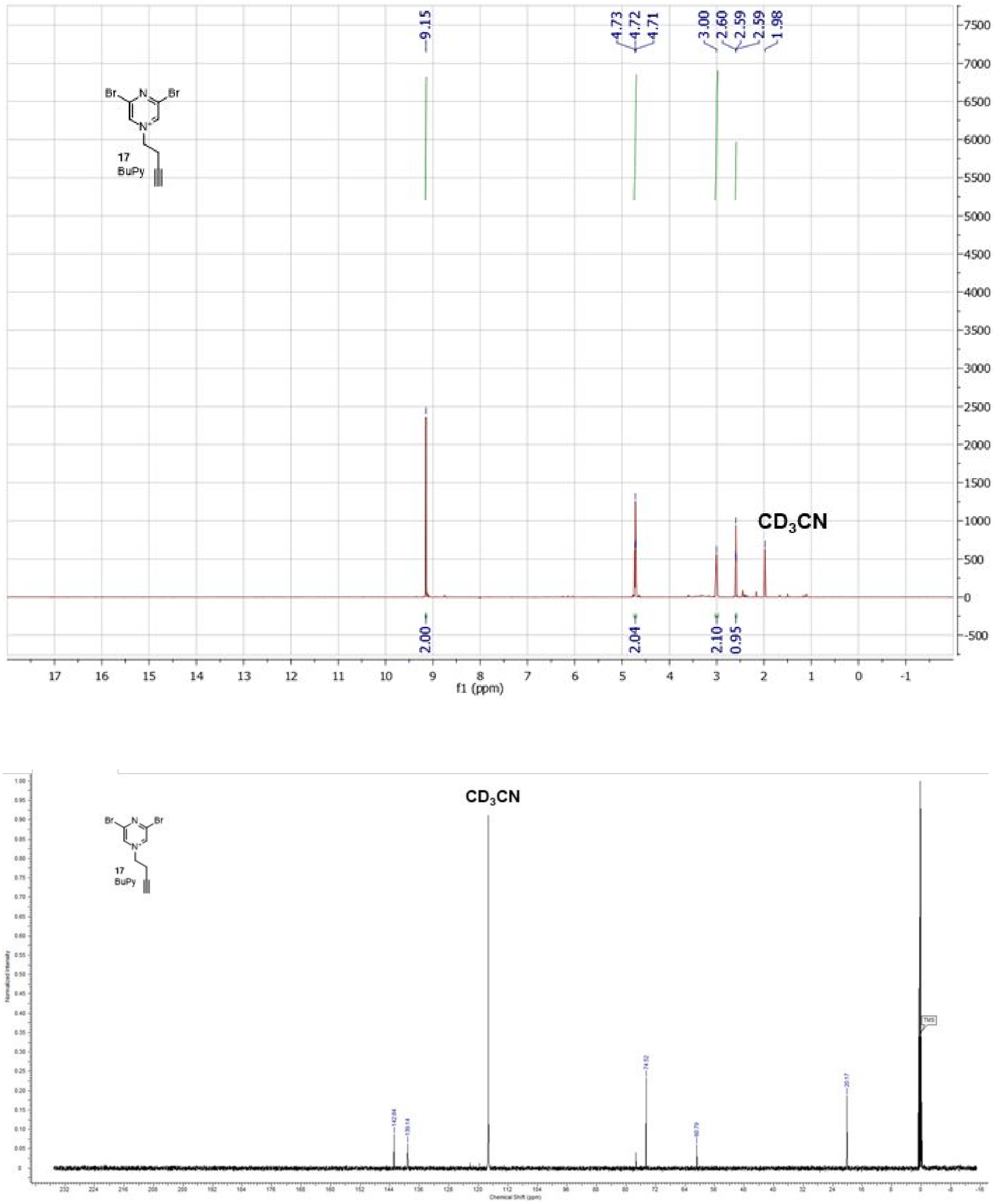

Figure S12. NMR spectra of **17** pyrazinium trifluoromethanesulfonate

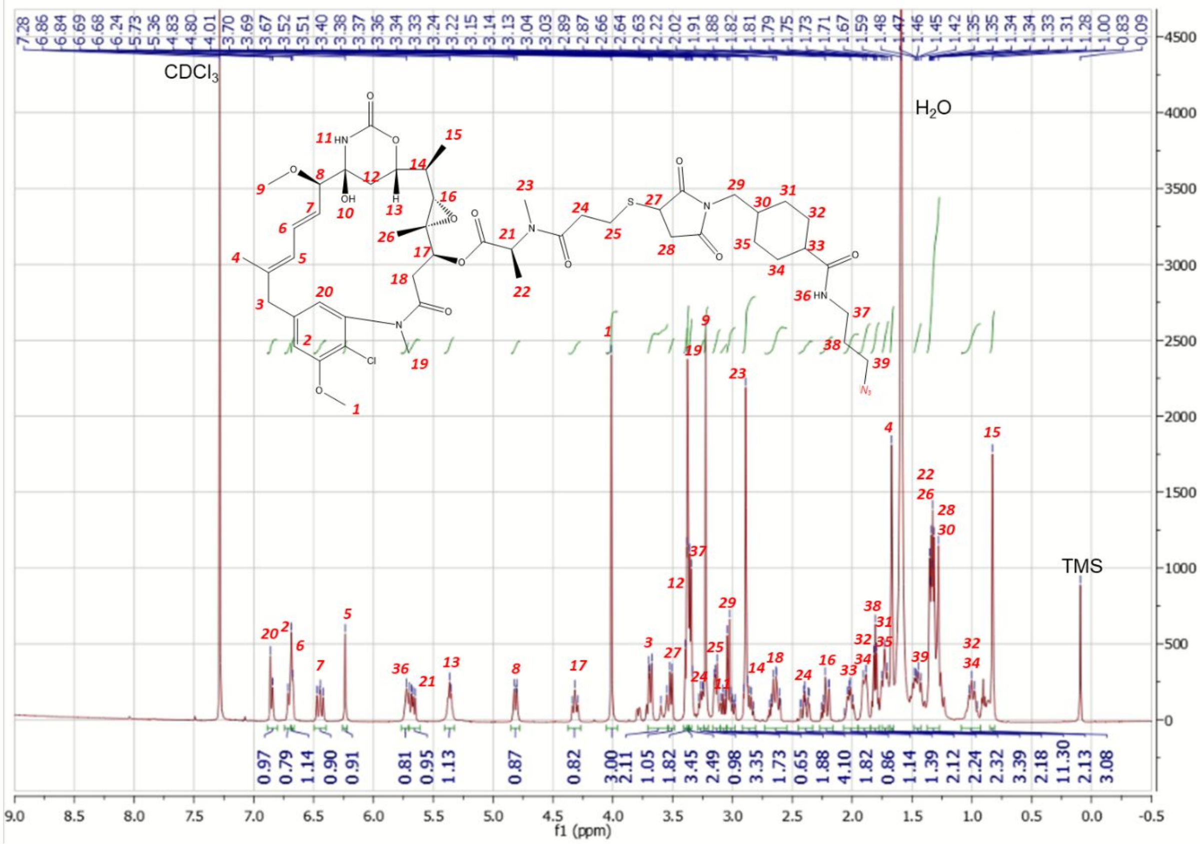

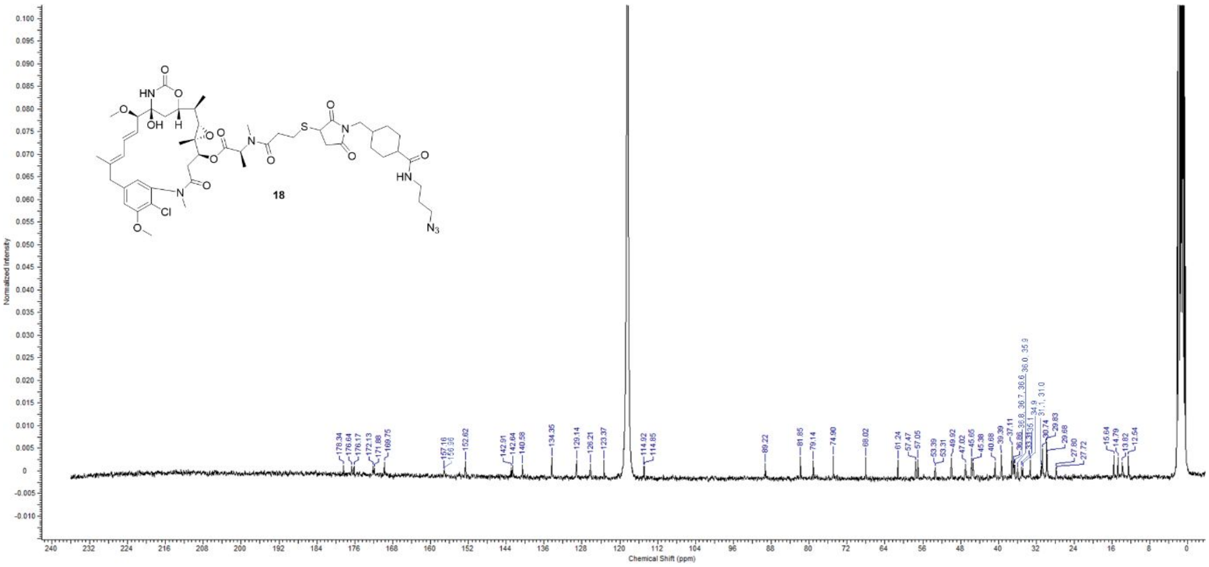

Figure S13. NMR spectra of **18** N_3-SMCC-DM1.

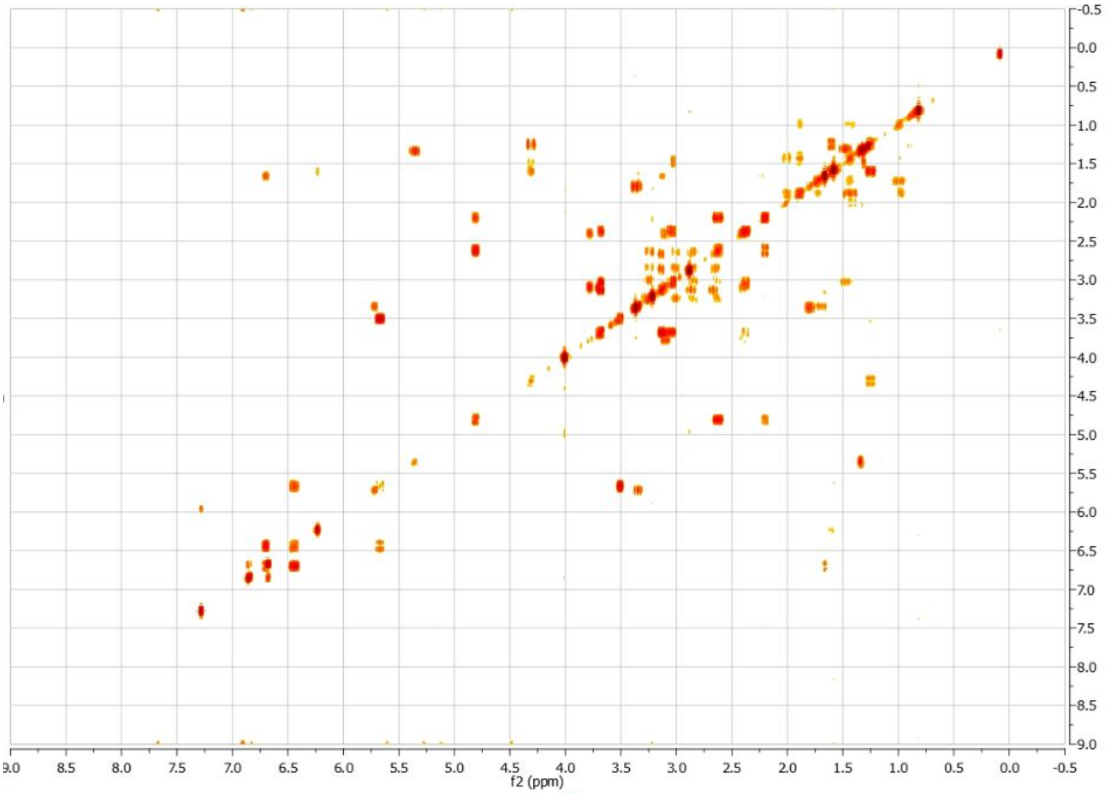

Figure S14. COSY 2D-NMR spectra of **18** N_3-SMCC-DM1

Supplementary References

[S1] H. Wang, J. Wu, L. Xu, K. Xie, C. Chen, and Y. Dong, "*Albumin nanoparticle encapsulation of potent cytotoxic therapeutics shows sustained drug release and alleviates cancer drug toxicity,*" Chem. Commun., vol. 53, no. 17, pp. 2618–2621, **2017**, doi: 10.1039/C6CC08978J.
